# Effects of abolishing Whi2 on the proteome and nitrogen catabolite repression-sensitive protein production

**DOI:** 10.1093/g3journal/jkab432

**Published:** 2021-12-17

**Authors:** Jennifer J Tate, Jana Marsikova, Libuse Vachova, Zdena Palkova, Terrance G Cooper

**Affiliations:** 1 Department of Microbiology, Immunology and Biochemistry, University of Tennessee Health Science Center, Memphis, TN 38163, USA; 2 Department of Genetics and Microbiology, Faculty of Science, Charles University, BIOCEV, 128 00 Prague, Czech Republic; 3 Institute of Microbiology of the Czech Academy of Sciences, BIOCEV, 142 20 Prague, Czech Republic

**Keywords:** TorC1 complex, Gln3, Whi2, nitrogen metabolism, synthetic complete medium, signal transduction, nuclear translocation, Gat1, DAL80, Gcn2

## Abstract

In yeast physiology, a commonly used reference condition for many experiments, including those involving nitrogen catabolite repression (NCR), is growth in synthetic complete (SC) medium. Four SC formulations, SC_CSH,1990_, SC_CSH,1994_, SC_CSH,2005_, and SC_ME_, have been used interchangeably as the nitrogen-rich medium of choice [Cold Spring Harbor Yeast Course Manuals (SC_CSH_) and a formulation in the methods in enzymology (SC_ME_)]. It has been tacitly presumed that all of these formulations support equivalent responses. However, a recent report concluded that (i) TorC1 activity is downregulated by the lower concentration of primarily leucine in SC_ME_ relative to SC_CSH_. (ii) The Whi2–Psr1/2 complex is responsible for this downregulation. TorC1 is a primary nitrogen-responsive regulator in yeast. Among its downstream targets is control of NCR-sensitive transcription activators Gln3 and Gat1. They in turn control production of catabolic transporters and enzymes needed to scavenge poor nitrogen sources (*e.g.*, Proline) and activate autophagy (*ATG14*). One of the reporters used in Chen *et al.* was an NCR-sensitive *DAL80-GFP* promoter fusion. This intrigued us because we expected minimal if any *DAL80* expression in SC medium. Therefore, we investigated the source of the Dal80-GFP production and the proteomes of wild-type and *whi2Δ* cells cultured in SC_CSH_ and SC_ME_. We found a massive and equivalent reorientation of amino acid biosynthetic proteins in both wild-type and *whi2Δ* cells even though both media contained high overall concentrations of amino acids. Gcn2 appears to play a significant regulatory role in this reorientation. NCR-sensitive *DAL80* expression and overall NCR-sensitive protein production were only marginally affected by the *whi2Δ*. In contrast, the levels of 58 proteins changed by an absolute value of log_2_ between 3 and 8 when Whi2 was abolished relative to wild type. Surprisingly, with only two exceptions could those proteins be related in GO analyses, *i.e.*, GO terms associated with carbohydrate metabolism and oxidative stress after shifting a *whi2Δ* from SC_CSH_ to SC_ME_ for 6 h. What was conspicuously missing were proteins related by TorC1- and NCR-associated GO terms.

## Introduction

Free living yeast cells face constantly changing nutritional environments. In response, they have evolved sophisticated mechanisms to successfully exploit times when nutrients are plentiful and tolerate those when they are not. In *Saccharomyces cerevisiae*, one of the principal mechanisms for sensing and responding to varying environmental conditions depends on the global nutrient-responsive protein kinase Target of Rapamycin Complex 1 (TorC1) (Beck and Hall 1999; Cardenas *et al.* 1999; Hardwick *et al.* 1999; Bertram *et al.* 2000). TorC1 is highly active when amino acids, in general and leucine, methionine, and/or glutamine in particular, are plenteous and inactive when they and the overall nitrogen supply are depleted (Bonfils *et al.* 2012; Sutter *et al.* 2013; Laxman *et al.* 2014; Stracka *et al.* 2014).

### TorC1 regulation

TorC1’s responses to changes in the nitrogen supply controls cell growth, and the downstream pathways associated with it. For example, when TorC1 is active, ribosome production, and the proteins needed to support translation as well as many other requisites for cell division are upregulated. Simultaneously, pathways that have evolved to deal with nutrient deprivation, *e.g.*, nitrogen scavenging, autophagy, and vacuolar nutrient mobilization, are severely down-regulated (Broach 2012; González and Hall 2017; Zhang *et al.* 2018; Hatakeyama and De Virgilio 2019a, 2019b).

**Figure 1:**
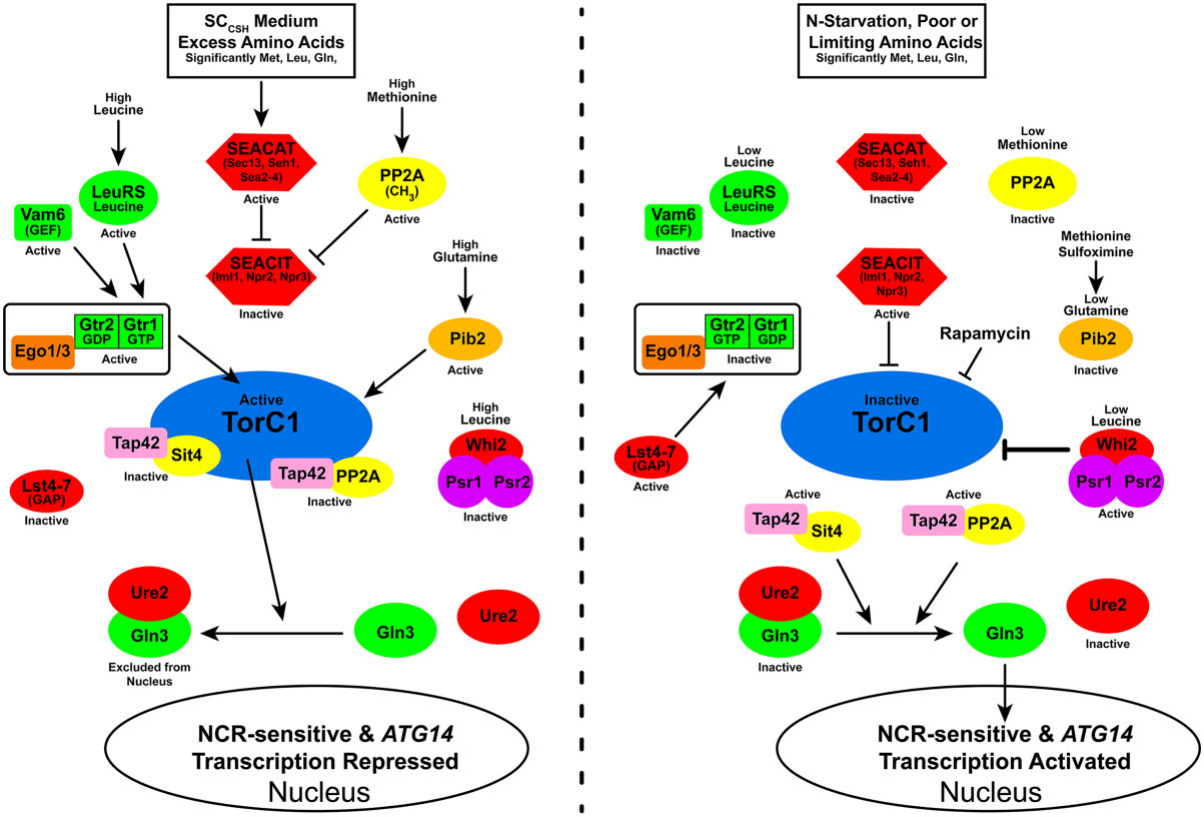
Diagram of the TorC1 regulatory pathway as it is generally viewed and Whi2 participation as reported (Chen *et al.* 2018). It is important to emphasize that “active” or “inactive,” as used in the figure, indicates overall activity. The designations may not apply to other functions or conditions that the depicted regulatory proteins may also be executing. For example, Sit4 functions both when TorC1 is highly active as well as inactive, *i.e.*, when complexed with Tap42 or not associated with Tap42; Sit4 and PP2A are both required along with Ure2 to maintain Gln3 in the cytoplasm in nitrogen-rich medium; TorC1 is rarely if ever fully inactive; Ego–Gtr complex components are required for nuclear Gln3 (Tate *et al.* 2015).

The detailed molecular mechanisms through which amino acids control TorC1 activity are being increasingly understood (Figure 1) (Binda *et al.* 2009, 2010; Bonfils *et al.* 2012; Sutter *et al.* 2013; Panchaud *et al.* 2013a, 2013b; Stracka *et al.* 2014; Peli-Gulli *et al.* 2015; Ukai *et al.* 2018; Hatakeyama *et al.* 2019; Hatakeyama and De Virgilio 2019a, 2019b; Hu *et al.* 2019). In amino acid excess, active GTP exchange factor Vam6 converts the Gtr components of the Gtr–Ego complex to their active Gtr1^GTP^–Gtr2^GDP^ form. This activated Gtr–Ego complex, in turn physically interacts with and activates TorC1 at the vacuolar membrane. Leucine tRNA synthetase (LeuRS) complexed with leucine also promotes Gtr1–GTP formation (Figure 1, left panel). TorC1 is also upregulated by Pib2 during high glutamine conditions. Further, glutamine, interacting with Pib2, activates TorC1 in a purified *in vitro* system (Tanigawa *et al.* 2021).

TorC1 activity is additionally regulated by three important complexes: SEACAT, consisting of Sec13, She1, Sea2-4; SEACIT, consisting of Iml1, Npr2, and Npr3; and methylated PP2A (Figure 1, left panel). In excess nitrogen, SEACAT inhibits SEACIT. As a result, the inactive form of SEACIT is unable to regulate TorC1 activity. SEACIT is additionally inhibited by methylated PP2A, which occurs when methionine and hence *S*-adenosyl methionine concentrations are high.

As amino acid supplies dwindle, the GTPase activating protein (GAP) complex, Lst4-Lst7, converts the Gtr complex to its inactive Gtr1^GDP^–Gtr2^GTP^ form (Figure 1, right panel). SEACAT is no longer able to inhibit the actions of SEACIT, which now downregulates TorC1. Low leucine, methionine, and glutamine additionally lowers the activities of the positive TorC1 regulators: LeuRS, PP2A, and Pib2.

### Nitrogen catabolite repression-sensitive regulation

The nitrogen scavenging and autophagy gene systems are regulated downstream of TorC1 by nitrogen catabolite repression (NCR) (Cooper 1982, 2002, 2004; Hofman-Bang 1999; Magasanik and Kaiser 2002; Broach 2012; Ljungdahl and Daignan-Fornier 2012; Conrad *et al.* 2014; Swinnen *et al.* 2014; González and Hall 2017; Zhang *et al.* 2018).

NCR consists of shared regulation by both TorC1 and Gcn2, another well studied global amino acid-responsive kinase (Staschke *et al.* 2010; Tate *et al.* 2017). When TorC1 is active, the PP2A- and PP2A-like Sit4 phosphatases interact with TorC1-bound Tor-Associated Protein Tap42 (Figure 1) (Di Como and Arndt 1996; Jiang and Broach 1999; Wang *et al.* 2003; Yan *et al.* 2006; Rohde *et al.* 2008). In this form, the phosphatases are inactive. As a result, Gln3 and Gat1, the NCR-sensitive transcriptional activators, are sequestered in the cytoplasm bound to the negative regulator protein Ure2 (Courchesne and Magasanik 1988; Coffman *et al.* 1994; Blinder *et al.* 1996; Cunningham *et al.* 2000; Kulkarni *et al.* 2001; Carvalho and Zheng 2003). Cytoplasmic Gln3 sequestration not only requires Ure2, but also the unbound forms of Sit4 and PP2A and the Gln3 dephosphorylations they mediate in nitrogen-rich medium (Tate *et al.* 2018, 2019).

As nitrogen supplies are depleted, Gcn2 is activated and TorC1 inhibited, resulting in release and thereby activation of the Tap42–phosphatase complexes from TorC1 (Figure 1, right panel). The Tap42–phosphatase complexes in turn dephosphorylate Gln3 permitting it to enter the nucleus and support NCR-sensitive transcription of genes encoding the transport and catabolic enzymes needed to scavenge poor nitrogen sources such as proline or allantoin. Gln3 also activates *ATG14* expression required for autophagy (Chan *et al.* 2001).

### Conditions that influence Gln3 regulation

There are five experimental conditions that elicit NCR-sensitive gene expression (Tate and Cooper 2013). Each condition has a specific requirement for the Sit4 and PP2A phosphatases: nitrogen limitation, *i.e.*, growth in poor nitrogen sources, *e.g.*, proline (Sit4); short-term nitrogen starvation (1–4 h depending on the strain background) (Sit4); long-term nitrogen starvation independent of, but associated with, G-1 arrest (>6 h) (neither phosphatase); treating cells growing in nitrogen rich medium with the TorC1 inhibitor rapamycin (PP2A and Sit4); or the glutamine synthetase inhibitor methionine sulfoximine (Msx) (neither phosphatase).

### Whi2-mediated regulation

When leucine, and other less defined amino acid, concentrations are low, a second amino acid-responsive protein complex, Whi2–Psr1/Psr2 has been reported to negatively regulate TorC1 activity in a SEACIT-Gtr- and PKA-independent manner (Figure 1, right panel). Whi2 was originally identified in a genetic screen of very small (wee) cells that continued to divide rather than G1 arrest as cultures transitioned into stationary phase (Carter and Sudbery 1980). Whi2, in association with the plasma membrane bound Psr1/2 phosphatases, were reported to dephosphorylate Msn2 thereby activating a general stress response (Martinez-Pastor *et al.* 1996; Kaida *et al.* 2002). This correlates with the observations that loss of Whi2 elicits hypersensitivity to heat, oxidative stress, and acetic acid.

In colonies growing on complete respiratory medium GM, Whi2 (together with Psr1 and Psr2) is involved in cell cooperation. The absence of Whi2 leads to competitive superiority via the mechanism of interference competition, likely due to increased production of an inhibitory metabolite. This Whi2–Psr1/Psr2 function is not associated with TORC1, *i.e.*, TorC1 is active in both wild-type and *whi2Δ* cells under the conditions assayed (Maršíková *et al*. 2020).

### Whi2 regulation in high and low amino acids

More recently, an exciting phenotype of *whi2* mutants was discovered fortuitously when their growth was compared with that of wild-type cells provided with either of two routine formulations of nitrogen-rich synthetic complete (SC) medium: one published in the 1994 and 2005 Cold Spring Harbor manuals (SC_CSH_) and the other in methods of enzymology (SC_ME_) (Guthrie and Fink 1991; Kaiser *et al.* 1994; Amberg *et al.* 2005). The main difference between these media is the overall concentrations of amino acids, particularly leucine; SC_ME_ medium has ∼30% lower concentrations of amino acids and >10-fold less leucine than SC_CSH_. The *whi2Δ* mutant cells were observed to grow much better than wild type in the lower amino acid medium. In a genome-wide survey of BY4741 knockout strains, *npr2Δ* and *npr3Δ* mutants were identified along with the *whi2Δ* based on this phenotype, *i.e.*, mutants exhibiting limited growth during nitrogen starvation, but more robust growth in low amino acid medium (Teng *et al.* 2013, 2018; Teng and Hardwick 2019). The identification of these three strains among the knockouts was important because, as noted above, Npr2 and Npr3 are components of the SEACIT inhibitor of TorC1 activity.

Subsequent studies of the *whi2Δ* led to the conclusions that Whi2: (i) is required to dampen TorC1-dependent cell growth and division as amino acid nitrogen decreases, but is not exhausted, and (ii) is a highly conserved inhibitor of TorC1 in response to low amino acids, particularly leucine (Teng *et al.* 2018; Teng and Hardwick 2019). However, low concentrations of leucine alone are insufficient to elicit Whi2-mediated TorC1 inhibition, low concentrations of other amino acids are required as well for inhibition to occur. Whi2 regulation of TorC1 appears to be restricted to amino acid sensing as the loss of Whi2 did not affect TorC1 inhibition elicited by low glucose (Chen *et al.* 2018).

Three widely used assays were employed to assess the effect of Whi2’s ability to inhibit TorC1 activity in SC_CSH_ and SC_ME_ media: (i) suppressed phosphorylation of the TorC1 target small ribosomal subunit protein Rps6, (ii) suppressed phosphorylation of Npr1 protein kinase, and (iii) expression of an NCR-sensitive reporter construct (Chen *et al.* 2018). The reporter employed was a *DAL80-GFP* promoter fusion plasmid in which GFP expression was driven by a *DAL80* promoter. The latter reporter was also used by Neklesa and Davis (2009) to identify *npr2* and *npr3* deletions (Rousselet *et al.* 1995). A Gat1–GFP assay was also used, along with a rapamycin control in recent studies investigating the regulation and dynamics of the expansion of papillae that arise during colony aging (Maršíková *et al*. 2020).

### Present study

The *DAL80-GFP* results obtained in the Whi2 study were surprising to us because both SC media contain highly repressive amounts of nitrogen. NCR-sensitive regulation has never, to our knowledge, been compared in SC_CSH_ and SC_ME_ media because these media, when used, are the negative control conditions for NCR-sensitive expression experiments. Therefore, our objective was to obtain a greater understanding of what occurred at the protein level when wild-type and *whi2Δ* cells were grown in or downshifted from the higher amino acid containing SC_CSH_ to lower containing SC_ME_ medium.

The data obtained demonstrate the major change in the proteome when cells are transferred from SC_CSH_ to SC_ME_ medium is a dramatic shift in amino acid metabolism, including many proteins that participate in amino acid biosynthesis even though SC_CSH_ and SC_ME_ media contain a high concentration (0.5%) of ammonia and 0.12% or 0.176% amino acids, respectively. Further, Whi2 presence or absence had only a marginal effect on overall NCR-sensitive gene expression. The *DAL80* (*Dal80-GFP*) expression, observed by Chen *et al.* (2018) likely derived from the fact *DAL80* transcription is strongly activated by Gat1 whose production is autogenously regulated and somewhat insensitive to NCR. In contrast to Dal80, there were far stronger effects on the levels of many other proteins.

## Materials and methods

### Strains, plasmids, and culture conditions

The *S. cerevisiae* strains and the plasmids we used in this work are in Table 1. This strain background was selected for analysis so that data from the present study can be directly compared with past and future data. Transformants, prepared by the lithium acetate method (Ito *et al.* 1983), were used as soon as possible after transformation (5 or less days).

**Table 1:** Strains, plasmids and primers used in this work

Strain/plasmid	Genotype	Reference
**Strains**		
BY4742	*MATα, his3*Δ*1, leu2*Δ0*, lys2*Δ0*, ura3*Δ0	Euroscarf.de
P1^a^	*MATα, his3*Δ*1, leu2*Δ*0, lys2*Δ*0, ura3*Δ*0, WHI2*	Maršíková *et al.* (2020)
P1-whi2	*MATα, his3*Δ1*, leu2*Δ*0, lys2*Δ*0, ura3*Δ*0, whi2*Δ*::kanMX SM* (SM, smooth colony morphology)	Maršíková *et al.* (2020)
**Plasmids**		
pRR536^b^	Gln3_1-730_–Myc_13_ (full length wild type with native promoter)	Rai *et al.* (2013, 2014)
pKA62^b^	Gat1_1-510_–Myc_13_ (full length wild type with native promoter)	Kulkarni *et al.* (2006)
**Primers**		
*DAL80*	5′-CCCACGTGCCAGAATTGTTT-3′	Georis *et al.* (2009)
	5′-TCAAGCTGATAGGCCTTGGT-3′	
*TBP1*	5′-TATAACCCCAAGCGTTTTGC-3′	Georis *et al.* (2009)
	5′-GCCAGCTTTGAGTCATCCTC-3′	

a BY4742 clone.

b Plasmids contain URA3 as the selectable marker.

Cultures (50 ml) were grown to mid-log phase (*A*_600 nm_ ∼ 0.5) in yeast nitrogen base (YNB, without amino acids or ammonia; VWR Life Science AMRESCO) minimal medium containing the indicated nitrogen source (final concentration, 0.1%). Leucine (120 µg/ml), histidine (20 µg/ml), and lysine (40 µg/ml) were added as needed to cover auxotrophic requirements. SC cultures were grown in either SC Cold Spring Harbor (SC_CSH_) or SC methods in enzymology (SC_ME_) to the *A*_600__nm_ indicated in the figures. Cells were treated with 200 ng/ml rapamycin (Sigma) for 15 or 20 min or 2 mM Msx (Sigma) for 30 min (Georis *et al.* 2011).

### Gln3–Myc_13_ and Gat1–Myc_13_ localization and image processing

These methods are reproduced from Tate *et al.* (2021) with permission of the publisher. Cell collection and Gln3**–**Myc_13_ (and Gat1**–**Myc_13_) visualization by indirect immunofluorescence microscopy were performed as described (Feller *et al.* 2013; Tate *et al.* 2019). Microscopic images for presentation were prepared using Adobe Photoshop and Illustrator programs. Level settings (shadow and highlight only) were altered where necessary to avoid any change or loss in cellular detail relative to that observed in the microscope; changes were applied uniformly to the image presented and were similar from one image to another. Mid-tone, gamma settings were never altered. These processed images were used for illustrative presentation only, NOT for scoring Gln3**–**Myc_13_ intracellular distributions.

### Determination of intracellular Gln3–Myc_13_ or Gat1–Myc_13_ localization

These methods are reproduced from Tate *et al.* (2021) with permission of the publisher. Gln3**–**Myc_13_ intracellular localization was manually scored in 200 or more cells for each data point. Unaltered, primary .zvi image files viewed with Zeiss AxioVision 3.0 and 4.8.1 software were exclusively used for scoring purposes. Cells were classified into one of three categories: cytoplasmic (cytoplasmic fluorescent material only, red histogram bars), nuclear-cytoplasmic (fluorescent material appears in both the cytoplasm and colocalizing with DAPI-positive material, DNA, yellow bars), or nuclear (fluorescent material colocalizing only with DAPI-positive material, green bars). Representative “standard” images and detailed descriptions of these categories appear in Figure 2 of Tate *et al.* (2009). The precision of our scoring has been repeatedly documented (Tate *et al.* 2006, 2010; Rai *et al.* 2013, 2014). Standard deviations of data from independent experiments appear as error bars. Greatest variation was observed when Gln3**–**Myc_13_ was significantly localized to more than one cellular compartment.

**Figure 2:**
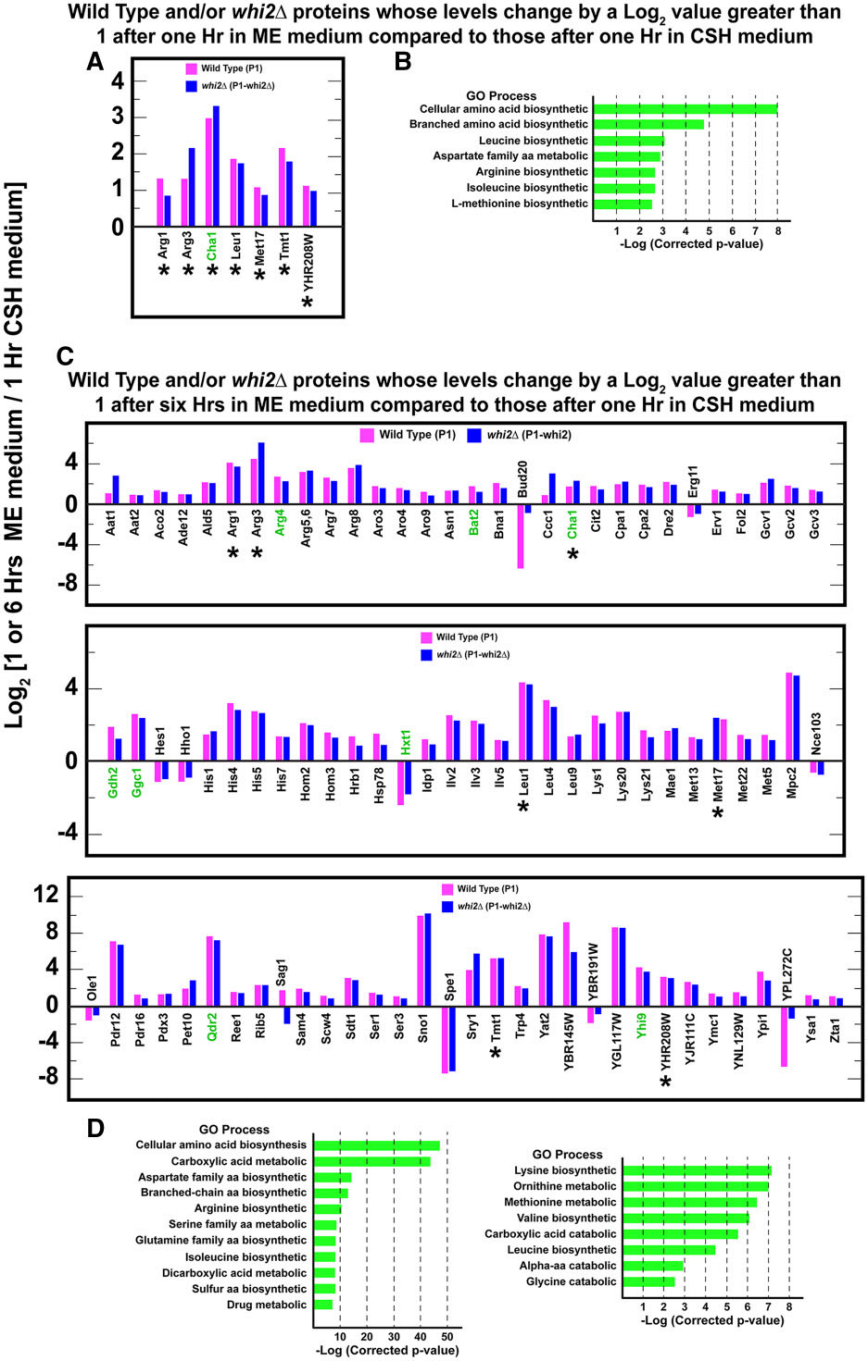
Proteins whose levels changed equal to or greater than absolute log_2_ values of 1, *i.e.*, twofold, in one or the other, *i.e.*, in wildtype (P1) and/or *whi2*Δ (P1-whi2), cells grown overnight in SC_CSH_ medium and then transferred to SC_ME_ medium for 1 h (A) or 6 h (C). Growth and transfer conditions are described in *Materials and Methods*. Proteins in wildtype and *whi2*Δ cells are designated in magenta and blue bars, respectively. Proteins that changed under both conditions are marked with an asterisk. Known or potentially NCR-sensitive proteins appear in green text. (B, D) SGD GO process analyses (using SGD GO term finder program) of the genes whose cognate proteins appear in (A) and (C), respectively. [*A corrected *P*-value is the smallest familywise significance level at which a particular comparison will be declared statistically significant as part of the multiple comparison testing.]

Images accompanying the histograms were chosen on the basis that they exhibited intracellular Gln3**–**Myc_13_ distributions as close as possible to those observed by quantitative scoring. However, identifying a field that precisely reflected the more quantitative scoring data were sometimes difficult unless the tagged protein was situated in a single cellular compartment.

### Cell collection for western blot or qRT-PCR analyses

These methods are reproduced from Tate *et al.* (2021) with permission of the publisher. Cultures to be analyzed were grown to mid-log phase (*A*_600__nm_ = 0.4–0.5) as described above. Once the desired *A*_600__nm_ was reached, or following treatment, the cells were harvested by filtration (using type HA, 0.45 mm Millipore filter), quickly scraped from the filter, placed in a sterile 1.5-ml microcentrifuge tube, and flash-frozen by submerging the microcentrifuge tube and cells in liquid nitrogen for 20–30 s. The total time for cell harvest to the point of submersion in liquid nitrogen was 25–35 s. The tube, still containing liquid nitrogen, was then quickly transferred to −80°C until further processing of the cells was performed.

### Protein extraction and western blot analyses

These methods are reproduced from Tate *et al.* (2021) with permission of the publisher. Extracts for western blots were prepared following the method of Liu *et al.* (2008). Total protein was extracted by lysing cells in a solution of 0.3 N NaOH, 1.2% β-mercaptoethanol (final concentrations), on ice for 10 min. Protein was then precipitated with trichloroacetic acid at a final concentration of 8%, for an additional 10 min on ice. Precipitated protein pellets were then resuspended in 1× sodium dodecyl sulfate (SDS) loading buffer and the extract neutralized with 1 M unbuffered Tris. Crude extracts were then boiled, protein resolved by SDS-PAGE (6% or 7% polyacrylamide) and transferred to nitrocellulose membrane (Bio-Rad) in non-SDS containing buffer.

Membranes were blocked for 1 h at room temperature with 5% Carnation milk in 1× TTBS (20 mM Tris–HCL pH 7.5, 0.05% Tween 20, 0.5 M NaCl). Membranes were then incubated overnight at room temperature with 9E10 (c-Myc) monoclonal antibody (sc-40; Santa Cruz Biotechnology) at a dilution of 1:1000 in 1× TBS (20 mM Tris–HCL pH 7.5, 0.5 M NaCl) plus 0.25% gelatin. Membranes were washed with 1× TBS and incubated with goat antimouse IgG (H + L)-horseradish peroxidase conjugate antibody (Bio-Rad) at a dilution of 1:10,000 for 1 h in 1× TBS containing 0.005% Tween 20 and 0.25% gelatin. Membranes were washed with 1× TBS containing 0.005% Tween 20 buffer. Immunoreactive species were detected using the SuperSignal West Pico Chemiluminescent Substrate kit (ThermoScientific) following the manufacturer’s instructions and results recorded on Classic blue autoradiography Film BX (Midwest Scientific).

### qRT-PCR analyses

These methods are reproduced from Rai *et al.* (2015) with permission of the publisher. Total RNA was extracted using the RNeasy Mini Kit (Qiagen), following the manufacturer’s instructions for purification of total RNA from yeast—mechanical disruption of cells. Two modifications were made to this protocol from our previous report: (i) cells were broken with glass beads (0.45 μm) using a BeadBug homogenizer (Benchmark Scientific): 4000 rpm, 4° for 30-s intervals followed by 30 s in an ice water bath, and (ii) on-column RNase-free DNase I treatment was performed for 1 h instead of 40 min. Quality of the total RNA was analyzed on an Agilent 2100 Bioanalyzer using the Agilent RNA 6000 Nanochip by the University of Tennessee Health Science Center (UTHSC) Molecular Resource Center. Complementary DNAs (cDNAs) were generated using the Transcriptor First Strand cDNA Synthesis Kit (Roche) following the manufacturer’s recommended protocol using both Oligo(dT)_18_ and random hexamer primers (provided with the kit) for synthesis. Samples were prepared for quantification with LightCycler 480 SYBR Green I Master Mix (KAPABiosystems) using the manufacturer’s protocol. Quantification and subsequent analysis of cDNAs were performed on a Roche LightCycler 480 Real Time PCR System using LightCycler 480 software version 1.5.

### Proteomic analyses

Comparison of proteomes was performed by nano LC–MS/MS analysis. Harvested cells were disrupted with glass beads (five times for 20 s in Fast-Prep, Thermo Savant) in 100 mM triethylammonium bicarbonate (TEAB), 10 mM Tris(2-carboxyethyl)phosphine, 50 mM chloroacetamide buffer containing 2% sodium deoxycholate; after the first two runs, samples were heated at 95°C for 5 min. Protein aliquots (30 µg per sample; determined by bi-quinchoninic acid assay, Sigma) were used for MS sample preparation. Samples were further processed with SP3 beads according to Hughes *et al.* (2019). Briefly, 5 µl of SP3 beads were added to 30 µg of proteins in lysis buffer and diluted to 50 µl with 100 mM TEAB. Protein binding was induced by adding ethanol to a final concentration of 60% (v/v). Samples were mixed and incubated for 5 min at laboratory temperature. Beads were washed two times with 180 µl of 80% ethanol and then samples digested with trypsin (trypsin/protein ratio 1/30), acidified with TFA to 1% final concentration. Peptides were desalted with C18 disks (Empore). Peptides (2 µg) from each sample were separated on nano-reversed-phase columns (EASY-Spray column a 50-cm × 75-mm ID, PepMap C18, 2 µm particles using a 1-h elution gradient and analyzed in DDA mode on a Orbitrap Fusion Tribrid mass spectrometer) (Thermo Scientific).

### Proteomic data analysis

Three biological replicates were analyzed for each strain and condition. Raw files were processed in MaxQuant (v.1.5.8.3) and checked against the latest version of the *S. cerevisiae* Uniprot database and the common contaminant database. Perseus (v.1.6.1.1) and Excel 2013 were used for further analysis. The significance of differences in protein abundance between two strains or conditions was determined using the unpaired two-tailed *t*-test. *P*-values of 0.05 or less were considered statistically significant. Functional categories enriched in specific proteome comparisons were identified using the GO term finder at SGD (https://www.yeastgenome.org/goTermFinder) (*P*-value 0.01). (Cherry *et al.* 2012; Ashburner *et al.* 2000; Gene Ontology Consortium 2021; Mi *et al.* 2019; GO version 0.86). The accession number for the mass spectrometry proteomic data set used in this work is PXD0280004 and may be found at http://www.ebi.ac.uk/pride/archive/projects/PXD028004.

## Results

This study was initiated in response to observations made by Chen *et al.* (2018). They reported that (i) GFP production supported by a *DAL80-GFP* plasmid was the same at 0, 3, and 6 h after a wild-type transformant was transferred (down-shifted) from a richer SC_CSH_ (synthetic complete Cold Spring Harbor recipe, Supplementary Table S1) to SC_ME_ (synthetic complete method in enzymology recipe) medium, the latter having a lower amino acid content, and (ii) a similar result, but at a 2/3 lower level when the experiment was repeated with a *whi2Δ* recipient (Chen *et al.* 2018; Supplementary Figure S3). The conclusion derived from these observations was that Whi2 regulated NCR-sensitive gene expression via its negative control of TorC1 activity. However, two characteristics of the reported data attracted our attention: (i) there was easily detectable GFP production in both nitrogen-rich media. In contrast, we expected GFP production to be undetectable in both highly nitrogen, repressive media because its production was being driven by an NCR-sensitive *DAL80* promoter, and (ii) in contrast to expectation, there was no successive increase in GFP production at 3 and 6 h after a downshift in which the cells were transferred from richer SC_CSH_ to poorer SC_ME_ medium. Therefore, our initial objective was to understand these two unexpected observations.

### Proteome in SC-grown wild-type and *whi2Δ* cells

Our ability to explain the differences between our expectations and the results of Chen *et al.* (2018) was limited by the fact that very little is known about the response of NCR-sensitive genes in such rich media for both wild-type and *whi2Δ* strains. This is because nitrogen replete conditions, such as those used in the experiments cited above, are routinely used as the negative controls for rapamycin addition or other types of experiments involving NCR-sensitive gene expression or protein production. Therefore, to better understand the detailed behavior of NCR-sensitive protein production in nitrogen rich media, we characterized the proteomes of wild-type and *whi2Δ* cells cultured in SC_CSH_ medium and then transferred to SC_ME_ medium for 1 or 6 h. These growth protocols were analogous to those used in the previous reports (Chen *et al.* 2018). We obtained data for 2,261 proteins and used a change of an absolute log_2_ value ≥1 as a significant change in protein levels. This criterion was not used in the gross comparison described in Figure 2 (Supplementary Tables S2 and S4). In this figure, and its associated tables, a change in one or the other wild-type and/or *whi2Δ* protein levels by an absolute log_2_ value ≥1 was sufficient for it to be included.

The levels of only seven proteins significantly changed and did so similarly in the wild-type and *whi2Δ* strains, 1 h after transferring the cells from SC_CSH_ to SC_ME_ (Figure 2A; Supplementary Table S2). These proteins were highly enriched for the gene ontology (GO) processes associated with amino acid biosynthesis (*P* < 0.01) (Figure 2B; Supplementary Table S3). In fact, six of the seven proteins catalyze steps in the biosynthesis of arginine, methionine, or the aliphatic branched chain amino acids leucine, isoleucine or valine (Supplementary Tables S2 and S3). This was remarkable because the cells were growing in amino acid rich media containing amounts of these amino acids normally added to minimal medium to cover arginine, leucine, or methionine auxotrophs.

The seventh protein, Cha1, is a serine/threonine-inducible, catabolic threonine/serine deaminase. This enzyme produces ammonia and pyruvate and is a central component of serine, glycine, and threonine catabolism. In this context, it is important to note that serine and threonine are the most abundant amino acids in SC_ME_ medium, *i.e.*, up to six times higher concentrations than found in SC_CSH_ medium (Supplementary Table S1; Figure 2B). The high concentrations of these amino acids may account for changes in the deaminase levels when cells are transferred from SC_CSH_ into SC_ME_ medium.

Six hours after the downshift to SC_ME_ medium, much more wide-spread changes occurred. The levels of 92 proteins differed in the two strains, including the seven whose levels increased at 1 h after down-shift (Figure 2C; Supplementary Table S4; common proteins in Figure 2, A and C are indicated with an asterisk). The highly enriched GO process terms that characterized these proteins were overwhelmingly associated with the biosynthesis of various amino acids (Figure 2D; Supplementary Table S5). This argued that shifting the cells from SC_CSH_ to SC_ME_ medium as well as nutrient utilization following the transfer elicited a dramatic reorientation of amino acid metabolism. In every case, proteins needed for amino acid biosynthesis increased. *A priori* one would have expected for some to increase while others decreased. Not only did the biosynthetic capabilities of the two strains change almost exclusively in a positive direction, but they also did so by nearly the same amounts whether in wild-type or *whi2Δ* cells.

The nature of the biosynthetic pathways involved was also informative. The changes were restricted to specific amino acids (Figure 2C; Supplementary Table S5). The most highly represented biosynthetic pathways in this experiment were those for arginine, histidine, lysine, serine, leucine, isoleucine, methionine, and aromatic amino acids. These amino acids correlate very well with those whose concentrations were decreased in SC_ME_ relative to SC_CSH_ medium (Supplementary Table S1, highlighted in yellow).

### Absence of NCR-sensitive proteins in proteome

Nitrogen/amino acid downshifts from rich to poor media result in the inhibition of TorC1 activity and corresponding increases in NCR-sensitive and autophagy gene expression (Tate *et al.* 2015; Tate and Cooper 2013; Figure 1). Therefore, based on the Chen *et al.* report, we expected to see significant representation of NCR-sensitive and autophagy-related proteins in our experiments. However, only three of the 92 proteins observed in cells transferred from richer SC_CSH_ to poorer SC_ME_ medium for 6 h derived from NCR-sensitive genes: Bat2, catabolic branched-chain amino acid aminotransferase; Gdh2, glutamate dehydrogenase catalyzing the conversion of glutamate to ammonia and α-ketoglutarate, and Yhi9, whose function is unknown but its loss results in a defective unfolded protein response (Figure 2C, green text).

**Figure 3:**
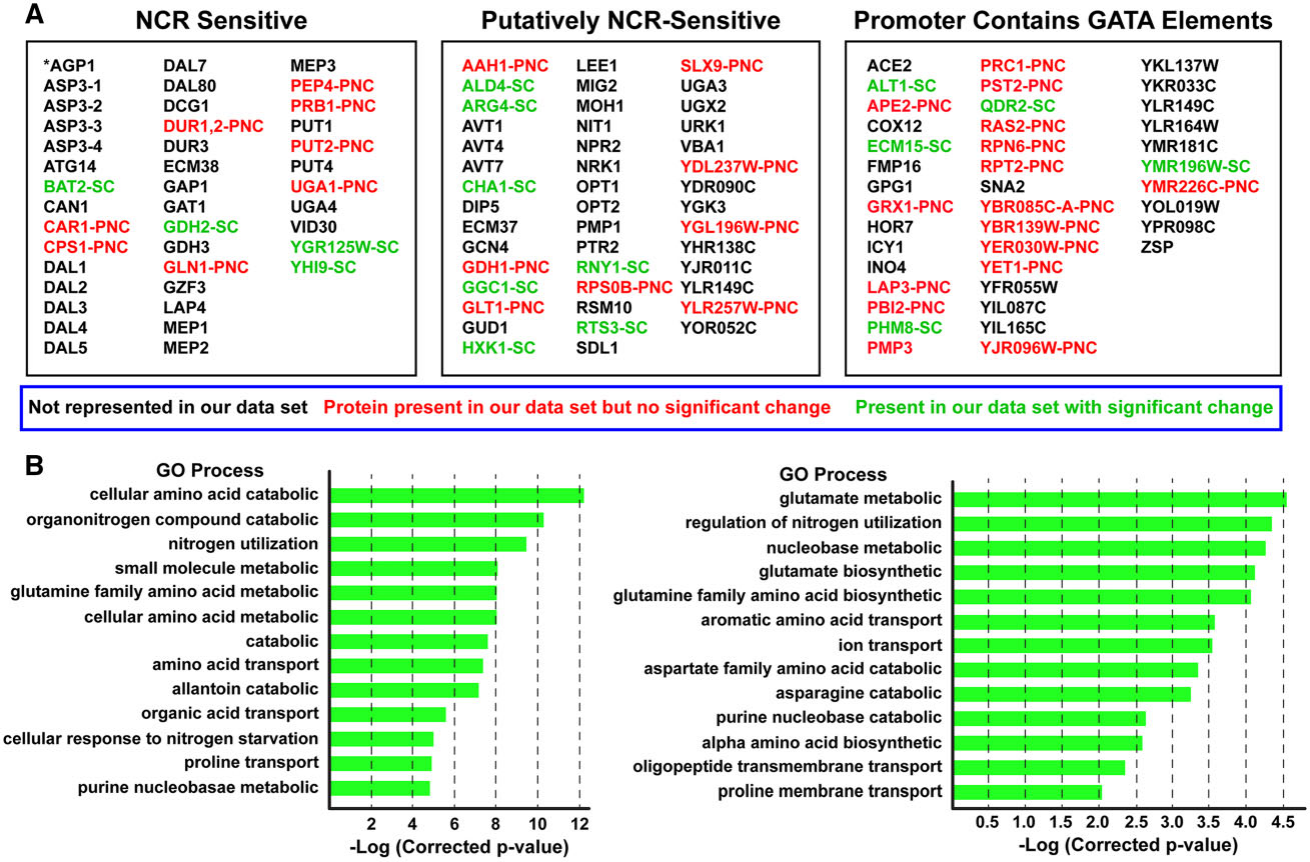
All known and potential nitrogen catabolite repression, NCR-sensitive genes in *Saccharomyces cerevisiae*. (A) Genes in black text were not represented in the proteomic data set. Genes in red text (labeled PNC, Present in proteomic data but No Change) were represented in the proteomic data set but their levels did not significantly change in any of the conditions we assayed during the course of all experiments we performed. Genes in green text (SC, Significant Change) underwent a significant change in one or more of the conditions we assayed during the course of experiments presented in this work. (B) GO process analysis output using the genes in (A) as the query. The GO list is incomplete. It is presented only to a −log(*corrected *P*-value) between 5 and 6 and 12.

One explanation for the paucity of NCR-sensitive proteins in the observed proteome might have been that we were overly stringent in defining this classification. This would not be surprising as it may have emanated from our and others experience with NCR-sensitive reporter genes where transcription data have been reported. Therefore, we generated a list of all known NCR-sensitive genes (41 genes), plus those classified as putatively NCR-sensitive (44 genes) and additionally even those genes containing GATA sequences in their promoters (40 genes). Recall that GATA sequences are the cores of the NCR-sensitive transcriptional activator (Gln3, Gat1) binding sites (Figure 3A; Supplementary Table S6) (Rai *et al.* 1989; Bysani *et al.* 1991; Scherens *et al.* 2006; Godard *et al.* 2007; Kontos *et al.* 2008; Georis *et al.* 2009; Ljungdahl and Daignan-Fornier 2012).

The highly enriched GO process terms associated with the 125 proteins we selected were those representative of nitrogenous compound transport, catabolism, and cellular responses to nitrogen or nutrient levels (Figure 3B; Supplementary Table S7). Note that we enlarged the number of GO terms to include subcategories in hopes of finding terms shared with those reported in Supplementary Table S5. If nitrogen catabolic processes were represented in Supplementary Table S5, the GO analyses in Supplementary Table S7 should have identified them. There was, however, little if any commonality in the GO process terms derived from the data in Figure 2 and possible NCR-sensitive genes (Figure 3). In short, we saw many fewer than expected NCR-sensitive protein changes even in the 6-h nitrogen downshifted cells we assayed.

The broadened classification did, however, increase the number NCR-related proteins in Figure 2C, but by only four: Arg4, argininosuccinate lyase, catalyzing the last step in arginine biosynthesis; Cha1, threonine/serine deaminase; Ggc1, a mitochondrial GTP/GDP transporter; and Qdr2, a DHA1 family antiporter. Further, only three of 19 highly enriched GO process terms were associated with catabolic processes (Figure 2D). Those catabolic processes are involved in C-1 metabolism, inter-conversion of amino acids and/or their precursors (Supplementary Tables S4 and S5). The involvement of C-1 metabolism was not surprising given the two and fourfold increase of threonine and serine in SC_ME_ medium.

If we consider all of the experiments in the present work *in toto*, the levels of only 16 proteins out of the expanded classification of 125 NCR-related genes changed: four demonstrably NCR-sensitive genes, seven putative NCR-sensitive genes and five additional GATA containing gene promoters (Figure 3A, green text). Additionally, proteins encoded by 32 NCR-related genes were represented in our data set, but their levels did not change (Figure 3A, red text).

One of the proteins that remained unchanged in our experiments has often been used as an indicator of cytoplasmic nitrogen limitation. It is the NCR-sensitive *CAR1* gene, encoding arginase which catalyzes the first step in arginine catabolism. Growing cells accumulate large amounts of arginine in their vacuoles (Zacharski and Cooper 1978). During times of cytoplasmic nitrogen limitation these arginine reserves are mobilized so that dividing cells can reach their G1 and G0 phases (Sumrada and Cooper 1978). The value of this capability is that G1 cells are orders of magnitude more resistant to environmental insults than are dividing cells (Schenberg-Frascino and Moustacchi 1972; Elliott and Futcher 1993). Therefore, the onset of a nitrogen downshift would also lead one to expect increased levels of arginase and the urea degradative protein urea amidolyase encoded by *DUR1,2* (Sumrada and Cooper 1978).

Neither of these proteins exhibited significant changes in our experiments (Figures 2, A and C and 3A). This result, however, can be viewed from another perspective. Mobilizing vacuolar arginine was unnecessary because arginine was present in both media at levels sufficient to cover a complete arginine auxotrophy. Further, it was being synthesized under the nitrogen replete conditions in SC_ME_ medium as evidenced by increases in arginine biosynthetic pathway proteins noted in Figure 2, A and C. In sum, these data argue that NCR-sensitive protein production was not a major target of Whi2–control and hence TorC1-mediated regulation when cells were growing in nitrogen replete conditions or even after a 6-h downshift to a lower but still significant nitrogen presence.

There was a second puzzling observation in the proteomic data. Gdh2, NAD glutamate dehydrogenase, which catalyzes the conversion of glutamate to ammonia, α-ketoglutarate and NADH, appeared in both wild-type and *whi2Δ* samples (Figure 2C). Why would the cells require ammonia production when the medium they were growing in contained 0.5% ammonia? Speculatively, one possibility is that ammonia production by Gdh2, under the conditions of our experiments, was required to maintain the cell’s redox balance by transferring reducing equivalents from NADPH to NAD, whereupon Gdh1 would quickly recycle the NADH, alpha-ketoglutarate, and ammonia back to glutamate. Alternatively, one may question to what extent would the *MEP* genes, encoding the ammonia transporters, be expressed in such nitrogen-rich (amino acids plus ammonia) media (Dubois and Grenson 1979; Marini *et al.* 1994; Airoldi *et al.* 2016)?

### Wild-type and *whi2Δ* cells respond indistinguishably in standard protocols assessing NCR-sensitive regulation

**Figure 4:**
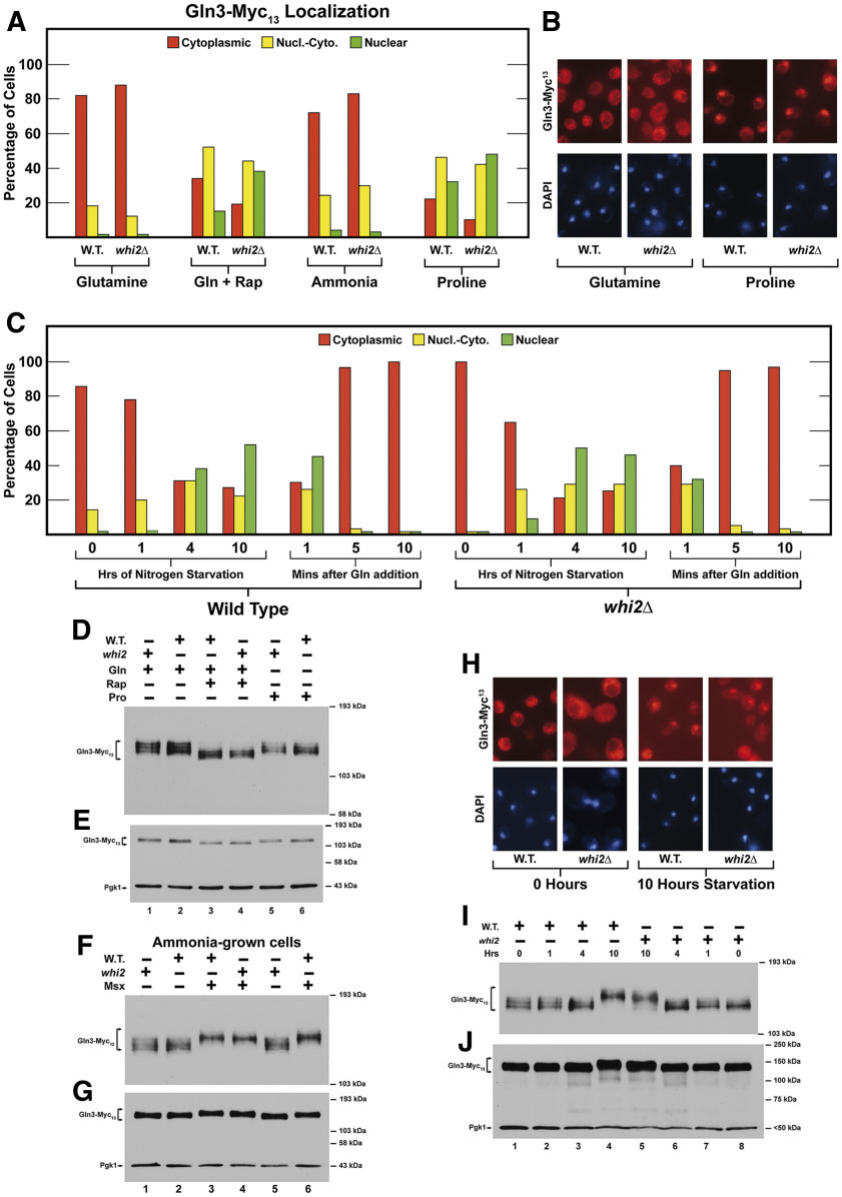
Responses of Gln3-Myc_13_ intracellular localization and phosphorylation to a *whi2*Δ. (A) Intracellular Gln3-Myc_13_ localization in wildtype (P1) and *whi2*Δ (P1-whi2) in cells provided with YNB–glutamine, ammonia, or proline as nitrogen source and with rapamycin added to glutamine (Gln + Rap) medium as described in *Materials and Methods* (our standard assay conditions, *N* = 1). Red histograms indicate Gln3-Myc_13_ located only in the cytoplasm, yellow indicates Gln3-Myc_13_ in both the cytoplasm and colocalizing with DAPI positive material, *i.e.*, nuclear-cytoplasmic and green indicates Gln3-Myc_13_ only colocalizing with DAPI positive material, *i.e.*, nuclear. Greater than two hundred cells were scored for each data point. (B, H) Illustrative examples of the types of cells that were scored in the three categories. (C) Response of Gln3-Myc_13_ in wild-type and *whi2*Δ cells undergoing short (1–4 h) and long term (10 h) nitrogen starvation followed by re-addition of glutamine for a short time. *N* = 1 because these results, which represent only a base line comparison, correlate with previously published experiments. (D) Gln3-Myc_13_ phosphorylation profiles in wildtype (P1) and *whi2*Δ (P1-whi2) cells provided with glutamine plus and minus rapamycin or proline. (F) Ammonia-grown cells untreated and treated with Msx. (I) Cultures were nitrogen starved for 0, 1, 4, and 10 h. (E, G, J) Duplicate blots to demonstrate the loading and transfer efficiencies.

Since less than half of the total number of *S. cerevisiae* proteins were represented in the data we obtained, one could argue that the Mep proteins escaped isolation or identification by the methods we used. Nonetheless, if Whi2 was downregulating TorC1, we had expected to see changes in many of the NCR-sensitive proteins. This was not the case. Cautious about deriving conclusions based on negative observations, we moved directly to investigate the transcription activators responsible for NCR-sensitive protein production, Gln3 and Gat1. In our standard nitrogen replete YNB–glutamine medium, Gln3**–**Myc_13_ was highly cytoplasmic in both the wild-type and *whi2Δ* (Figure 4, A and B). Cytoplasmic Gln3**–**Myc_13_ partially migrated into the nuclei of rapamycin-treated cells yielding a tripartite distribution of Gln3**–**Myc_13_ in all three of the scoring categories in both strains. Again, there was no detectable difference between wild-type and *whi2Δ* cells.

To evaluate possible Whi2 participation across the spectrum of NCR regulation, we extended this experiment to increasingly derepressive conditions using ammonia or proline as nitrogen source (Figure 4, A and B). Gln3**–**Myc_13_ responded only minimally in ammonia but similarly to rapamycin treated cells when proline was provided as the nitrogen source. Again, we were at a loss to convincingly argue that wild-type and *whi2Δ* cell responses were much different from one another. (Figure 4, A and B). In the most derepressive condition, *i.e.*, nitrogen starvation, wild-type, and *whi2Δ* cells also responded similarly (Figure 4C). In sum, we observed no demonstrable difference in the responses of wild-type and *whi2Δ* cells to any of the experimental conditions normally used to assess NCR-sensitivity.

### Gln3 phosphorylation profiles are the same in wild-type and *whi2Δ* cells

Gln3 is a highly phosphorylated protein whose intracellular localization and function are highly influenced by its phosphorylation (Cox *et al.* 2004; Tate *et al.* 2009, 2010; Georis *et al.* 2011; Rai *et al.* 2014; Tate *et al.* 2019). Further, protein phosphorylation (Rps6 and Npr1) was convincingly shown to be affected by deletion of *WHI2* (Chen *et al.* 2018). Therefore, we followed Gln3**–**Myc_13_ phosphorylation under conditions where its phosphorylation profiles were known to change. The responses of Gln3**–**Myc_13_ phosphorylation to rapamycin and Msx in wild-type cells were indistinguishable to those observed in the *whi2Δ* (Figure 4, D–G). The only possible differences we observed were perhaps slightly higher Gln3**–**Myc_13_ phosphorylation in unstarved wild-type cells and those nitrogen starved for 10 h compared with those in the *whi2Δ* (Figure 4, I and J). The lack of Whi2-dependent alterations in Gln3**–**Myc_13_ phosphorylation contrasted markedly with those observed by rapamycin treatment or deletion of the *SIT4* and *PPH21/22* (PP2A) phosphatase genes (Beck and Hall 1999; Bertram *et al.* 2000; Cox *et al.* 2004; Tate *et al.* 2009, 2010, 2019; Georis *et al.* 2011; Rai *et al.* 2014).

### Gln3 localization in high and low amino acids is indistinguishable in wild-type and *whi2Δ* cells

**Figure 5:**
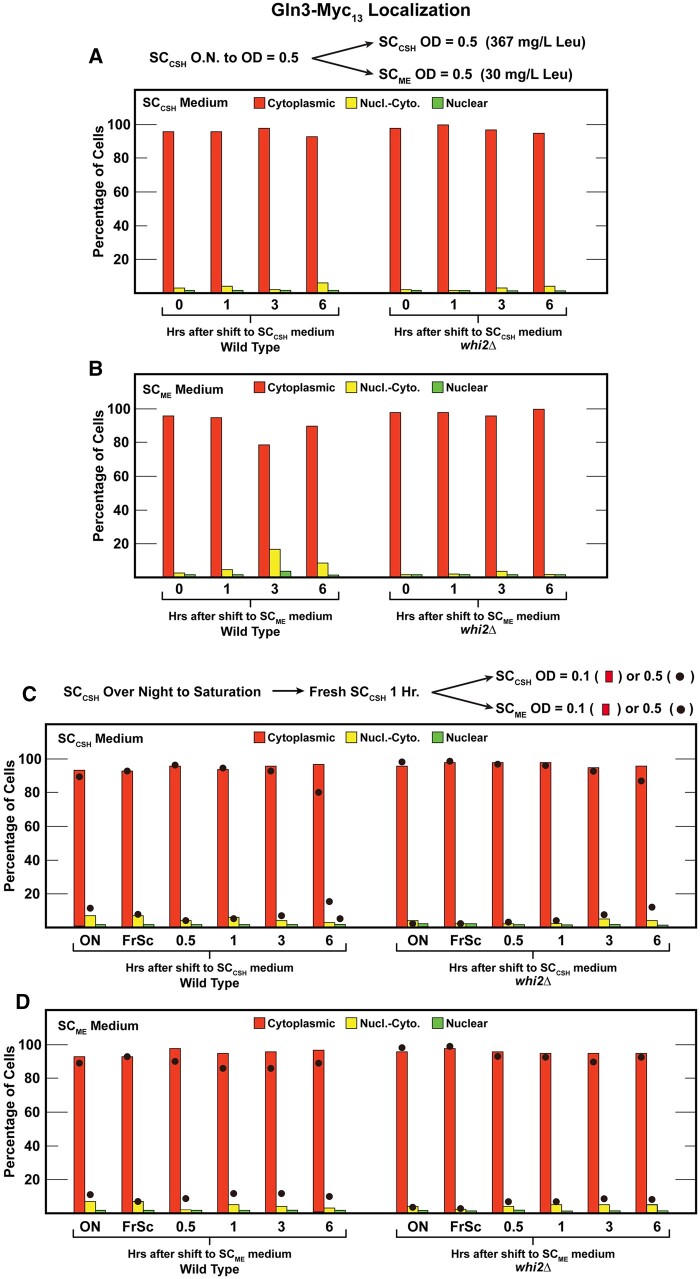
Gln3-Myc_13_ localization in wildtype (P1) or *whi2*Δ (P1-whi2) cells grown according to protocols reported by Chen *et al.* (2018). (A, B) Cultures were pre-grown overnight in SC_CSH_ medium (contains 367 mg/l leucine) to a cell density of *A*_600 nm_=0.5. The cultures were then split with one half transferred back into fresh SC_CSH_ medium and the other half transferred to fresh SC_ME_ medium (contains 30 mg/l leucine) for 0–6 h; both cultures to a cell density of *A*_600 nm_=0.5. (C, D) Cultures were pregrown overnight to saturation in SC_CSH_ medium (ON) and then transferred to fresh SC_CSH_ medium for 1 h (FrSC). Each of the cultures was then split. One half of the cultures were resuspended to a cell density of *A*_600 nm_ = 0.1 (the histogram) or *A*_600 nm_ = 0.5 (filled circles) in SC_CSH_ or SC_ME_ medium for 0–6 h. The experiments in (A) and (B) represent data from different cultures performed on different days than those in (C) and (D). We did not repeat these experiments because all four panels are variations of the same experiment and all gave identical results.

Concerned our results might derive from technical differences between our routine protocols and those previously reported, we repeated, as closely as we could, the conditions used in the Chen *et al.* (2018) report. First, we cultured wild-type and *whi2Δ* cells overnight in SC_CSH_ medium to an *A*_600__nm_ = 0.5. The culture was then split and transferred to either SC_CSH_ or SC_ME_ medium, as Chen *et al.* (2018) had used, and Gln3**–**Myc_13_ localization followed for an additional 6 h (Figure 5, A and B). Again, the results obtained with wild type and *whi2Δ* were indistinguishable. Gln3**–**Myc_13_ was almost completely cytoplasmic throughout the experiment.

Additionally, Chen *et al.* (2018) pregrew their cells to saturation in SC_CSH_ medium, transferred them to fresh SC_CSH_ medium for 1 h and then transferred them a second time to either SC_CSH_ or SC_ME_ media. We could not ascertain the cell number used following the second transfer in Chen *et al.*’s experiments, and so we performed our experiments with both low (*A*_600__nm_ = 0.1) and higher (*A*_600__nm_ = 0.5) cell numbers. Behaviors of the two strains were again indistinguishable (Figure 5, C and D). Histograms in these figures represent data with the low cell number, whereas the filled circles represent those obtained with the higher cell number.

### Gat1 NCR-sensitivity is indistinguishable in wild-type and *whi2Δ* cells

The above experiments with Gln3 lead us to conclude that Whi2 was not playing a demonstrable role in the regulation of its intracellular localization and hence its transcriptional function. There is, however, a second GATA-family transcription activator, Gat1, whose regulation differs somewhat from that of Gln3 (Kulkarni *et al.* 2006; Georis *et al.* 2008, 2011). For example, while Gat1 localization and function are nitrogen-responsive, its production is autogenous, its intracellular localization is not as NCR-sensitive as that of Gln3, even though it is more highly TorC1-regulated (rapamycin-responsive).

**Figure 6:**
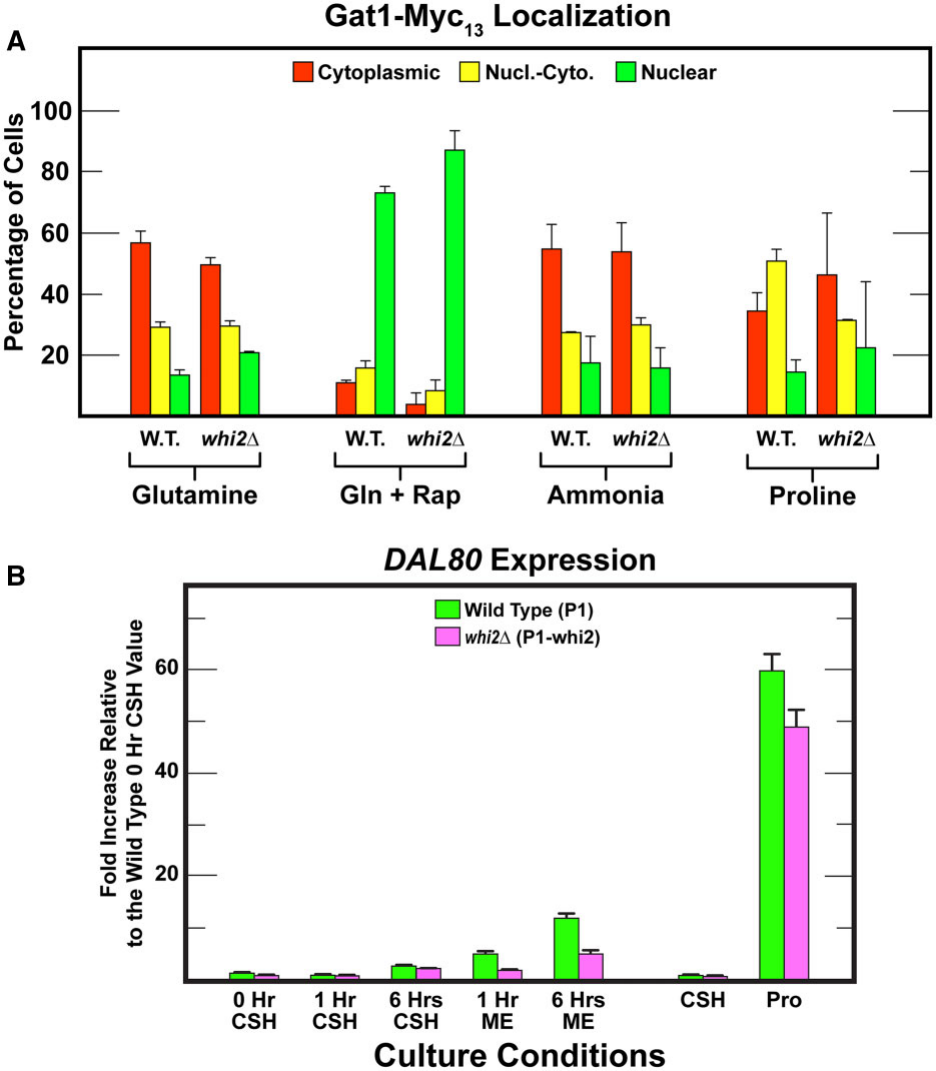
(A) Responses of Gat1–Myc_13_ intracellular localization in wildtype (P1) and *whi2*Δ (P1-whi2) cells provided with YNB–glutamine, ammonia, or proline as nitrogen source and with rapamycin added to glutamine (Gln + Rap) medium as described in *Materials and Methods* (our standard assay conditions). Data presentations are as described in Figure 3. (B) qPCR measurements of *DAL80* expression in wild type (P1, green bars) and *whi2*Δ (P1-whi2, magenta bars) in cells grown for 0, 1, and 6 h in either SC_CSH_ or SC_ME_ medium. Cells were also cultured in SC_CSH_ and YNB–proline (Pro) to demonstrate *DAL80* expression in response to nitrogen catabolite repression.

Therefore, we compared the responses of Gat1–Myc_13_ localization in wild-type and *whi2Δ* transformants. In contrast with Gln3**–**Myc_13_, Gat1–Myc_13_ exhibited less cytoplasmic sequestration in nitrogen replete glutamine medium (Figure 6A). However, wild-type and *whi2Δ* cells again responded similarly. Gat1 also exhibited a much stronger response to rapamycin treatment than Gln3**–**Myc_13_, becoming largely nuclear in both wild-type and *whi2Δ* cells (Figure 6A). The strong rapamycin response suggested the lack of Whi2 might have a stronger effect on Gat1–Myc_13_ localization as NCR was reduced. However, this was not observed. Gat1–Myc_13_ distributions in ammonia-grown wild-type and *whi2Δ* cells did not differ greatly from those with glutamine (Figure 6A). There was a modest nuclear shift of Gat1–Myc_13_ in proline medium, but again wild-type and *whi2Δ* cells did not yield convincingly different results. This indicated, importantly, that Gat1–Myc_13_, unlike Gln3**–**Myc_13_ localization was exhibiting little if any demonstrable NCR-sensitivity in this strain background even though it positively responded to rapamycin addition.

### Gat1 responds to shift from high to low amino acids at different rates in wild-type and *whi2Δ* cells

**Figure 7:**
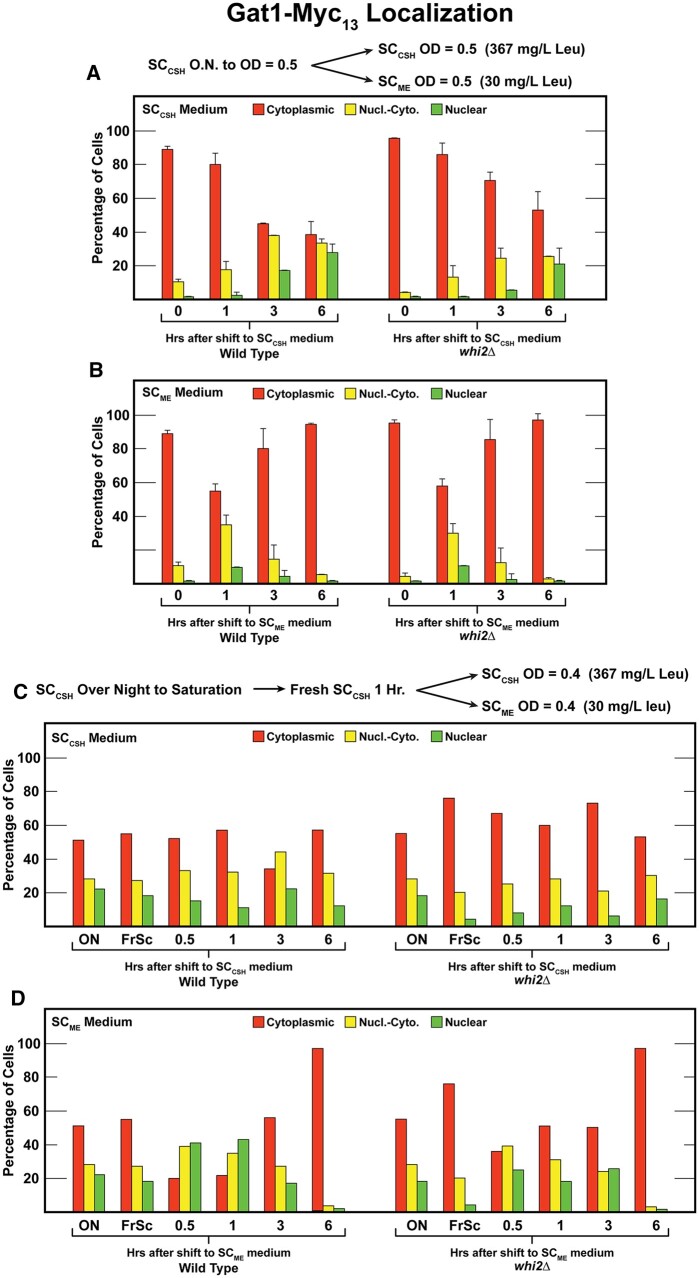
Gat1–Myc_13_ localization in wildtype (P1) or *whi2*Δ (P1-whi2) cells grown according to protocols reported by Chen *et al.* (2018). (A, B) Cultures were pre-grown overnight in SC_CSH_ medium (contains 367 mg/l leucine) to a cell density of *A*_600 nm_=0.5. The cultures were then split with one half transferred back into fresh SC_CSH_ medium and the other half transferred to fresh SC_ME_ medium (contains 30 mg/l leucine) for 0–6 h; both cultures to a cell density of *A*_600 nm_=0.5. (C, D) Cultures were pregrown overnight to saturation in SC_CSH_ medium (ON) and then transferred to fresh SC_CSH_ medium for 1 h (FrSC). The cultures were then split and then transferred the cells to fresh SC_CSH_ and SC_ME_ media at a starting *A*_600 nm_=0.4 for 30 min, 1, 3, and 6 h. Experiments in (A) and (B) represent data from different cultures (biological replicates, *N* = 2) analyzed on different days than those in (C) and (D) [*N* = 1, because data are overall consistent with those obtained in (A) and (B)].

We again argued that our standard assay conditions might not yield the same results as those reported by Chen *et al.* Therefore, we performed two more experiments using Chen *et al.*’s protocols (Figure 7). The main difference between results with wild-type and *whi2Δ* cells was the speed with which Gat1–Myc_13_ began migrating into the nucleus after the shift from SC_CSH_ to SC_CSH_ medium (Figure 7A). A greater fraction of Gat1–Myc_13_ relocated to the nuclei of wild-type cells at 3 h after the shift. This did not occur in the *whi2Δ* mutant until 6 h and even then, cytoplasmic Gat1–Myc_13_ remained higher than in the wild type. One could argue that these results are consistent with a loss of Whi2 diminishing TorC1 downregulation. This, in turn, would result in higher Gat1–Myc_13_ cytoplasmic sequestration in the *whi2Δ*. Importantly, however, this difference did not occur when cells were shifted from the SC_CSH_ to SC_ME_ medium with its lower concentrations of amino acids and particularly leucine (Figure 7B). Wild-type and *whi2Δ* cells responded indistinguishably to this transfer. Unfortunately, we can offer no speculation about why the shift into SC_ME_ medium for increasing amounts of time yielded largely cytoplasmic Gat1–Myc_13_ sequestration. *A priori*, we would have expected just the opposite, *i.e.*, a greater response as cells spent increasing times in the SC_ME_ medium.

Finally, analogous to the experiment reported by Chen *et al.*, we cultured wild-type and *whi2Δ* cells to saturation in SC_CSH_ medium (Figure 7, C and D), transferred them to fresh SC_CSH_ medium (FrSC) for 1 h and then split each of the cultures and transferred them a second time to either SC_CSH_ or SC_ME_ medium (Figure 7, C and D, respectively). Shifting the saturated cultures from overnight incubation in SC_CSH_ to fresh SC_CSH_ medium for 1 h resulted in consistently more cytoplasmic Gat1–Myc_13_ sequestration in the *whi2Δ* than wild type (Figure 7, C and D).

In the case of the SC_CSH_ to SC_ME_ shift, the intracellular distributions of Gat1–Myc_13_ in wild-type and *whi2Δ* cells were similar at the half hour time point (Figure 7D). In response to the SC_CSH_ to SC_ME_ shift, Gat1–Myc_13_ began translocating into the nucleus. However, this was a transient effect which ended at 3 h after the transfer in wild type and 1 h in the *whi2Δ*. By 6 h Gat1–Myc_13_ was efficiently sequestered in the cytoplasm of both strains. This is opposite of what one would *a priori* expect since the cells had been in the poorer of the two media downshifted for 6 h. The expectation was that the Gat1–Myc_13_ would be more nuclear at 6 h than earlier.

We are unable to speculate about the molecular mechanisms generating these unconventional results. We do, however, now have a possible explanation for the *DAL80-GFP* expression in Chen *et al.*’s experiments. *DAL80-GFP* production required the action of a GATA-family transcription activator. Gat1 provides this requirement. Further, Gat1 plays a very large role in *DAL80* expression (Cunningham *et al.* 2000). One may then justifiably query why Gat1 was even being produced since we clearly demonstrated that Gln3**–**Myc_13_ was securely sequestered in the cytoplasm of all experiments in SC_CSH_ and SC_ME_ media? *GAT1* expression like that of *DAL80* is partially Gln3-independent. This partial independence occurs because *GAT1* expression is autogenously activated and Gat1 significantly activates *DAL80* expression (Coffman *et al.* 1996).

### Dal80 protein production *vs DAL80* gene expression

Both our and the earlier experiments by Chen *et al.*, rested on a presumption, *i.e.*, Whi2 was a major negative regulator of TorC1 which in turn telegraphed its response to the downstream target genes. There was, however, an alternative way of viewing the data. What if NCR-sensitive protein production was not a major target of Whi2-mediated TorC1 regulation either because: (i) of the extent to which Whi2 downregulated TorC1 or (ii) TorC1 control of its downstream targets was distinctly target-dependent and finely graded? Either interpretation would have been consistent with both earlier reported and present results.

As noted above, *DAL80* is exquisitely activated by Gat1, more so than many other NCR-sensitive genes. What if that characteristic contributed to the preference of using the *DAL80-GFP* reporter both by Chen *et al.* (2018) and earlier experiments by Neklesa and Davis (2009)? In the Chen *et al.* experiment, GFP production was uniformly high in SC_CSH_ medium as well as 3 and 6 h after wild-type cells were transferred to SC_ME_ medium (see Supplementary Figure 3 in Chen *et al.*, 2018). When the experiment was repeated in the *whi2Δ*, GFP production was uniformly lower (approximately two to threefold) at 0, 3, and 6 h after downshifting the cells to SC_ME_ medium.

Since the *DAL80-GFP* plasmid was not available, we assayed *DAL80* expression directly (Figure 6B). In SC_CSH_ medium, *DAL80* expression was initially almost undetectable in both wild-type and *whi2Δ* cells (Figure 6B, 0 or 1 h in SC_CSH_ medium). After 6 h in SC_CSH_ medium, there was a small (two to threefold) increase in *DAL80* mRNA in the wild type which was not convincingly lower in the *whi2Δ* (Figure 6B 1 *vs* 6 h SC_CSH_). Note, however, that *DAL80* expression behaved predictably, *i.e.*, the longer the cells grew, depleting nitrogen as they did so, the more *DAL80* expression increased.

When cells were transferred from the richer SC_CSH_ to the poorer SC_ME_ medium for 1 and then 6 h, *DAL80* expression in the wild type increased approximately fivefold and 12-fold compared with approximately two and fivefold in the *whi2Δ*; these comparisons were relative to the 0-h SC_CSH_ control. However, the longer wild-type and *whi2Δ* cells were grown in the SC_ME_ medium the more *DAL80* expression increased. It is important to note that the degree to which *DAL80* expression was downregulated in *whi2Δ* cells was not dependent on the time that the cells spent in the poorer medium, as would normally be expected by the following reasoning. As the concentration of nitrogen decreased in the SC_ME_ medium from 1 to 6 h, so too would the level of TorC1 activation. In other words, TorC1 activation would be lower at 6 h than at 1 h. Correspondingly, the degree to which Dal80-GFP increased due to loss of Whi2 would also be expected to be greater at 6 than at 1 h. Experimentally, the reduction in *whi2Δ* cells was about twofold at both time points.

The above data were placed into proper perspective when we performed a standard *in vivo* assay for NCR-sensitivity. *DAL80* expression in derepressed proline-grown (Pro) cells was ∼60-fold higher than in the SC_CSH_ medium (Figure 6B). Further, abolishing Whi2 only modestly decreased (<18%) *DAL80* expression in the proline-grown cells. In other words, NCR-sensitive regulation is a minor target of Whi2. What remains unknown is whether Whi2 was acting directly or indirectly on TorC1. The uncertainty derives from the fact that the major physiological response observed in the proteomic data was a reorientation and upregulation of amino acid biosynthesis that (i) was independent of Whi2 and (ii) was highly correlated with the amino acids whose concentrations differed between the two SC media.

### Major protein targets altered only in wild-type cells shifted from SC_CSH_ to SC_ME_ medium

If NCR-sensitive gene expression was a minor target of Whi2-mediated regulation, what were Whi2’s major targets? The earlier proteomic data we discussed (Figure 2) focused only on the proteins whose levels changed in both wild-type and *whi2Δ* strains cultured in SC_CSH_ and SC_ME_ media. We additionally identified groups of proteins whose levels changed by absolute values of log_2_ >1 uniquely in a wild-type or *whi2Δ* strain after being transferred from SC_CSH_ to SC_ME_ medium. Indeed, unlike the NCR-sensitive proteins, these changes were dramatic.

**Figure 8:**
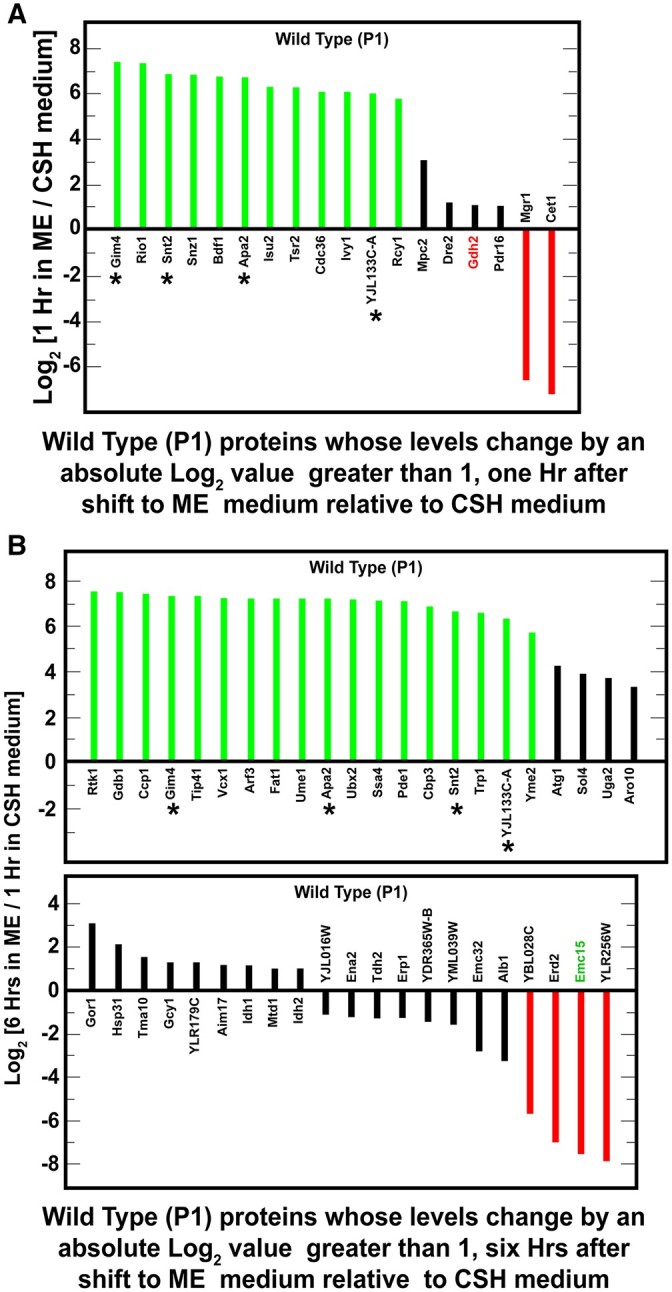
Proteins whose levels changed by absolute log_2_ values ≥1 (*i.e.*, equal to or greater than twofold) in wild-type (P1) cells but not in *whi2*Δ (P1-whi2) cells. Cells were cultured for 1 h (A) or 6 h (B) in SC_ME_ medium and the results compared with those obtained after cells were cultured for 1 h in SC_CSH_ medium. Prior to the beginning of the experiment, cells were pregrown overnight in SC_CSH_ medium to an *A*_600 nm_ = 0.35. Genes whose proteins changed at both 1 and 6 h are marked with an asterisk. NCR-sensitive genes appear in green text. SGD GO process analysis of the genes in (A) or (B) did not yield any significant results with *P*-value ≤0.01.

Twelve proteins increased by log_2_ values >5–6 in wild-type cells after 1 h in SC_ME_ relative to SC_CSH_ medium, thereby yielding positive values for log_2_ (1 h in SC_ME_/SC_CSH_ medium) (Figure 8A, green bars; Supplementary Table S8). In contrast only two proteins decreased by a log_2_ values <−6 1-h in these conditions thereby yielding negative values for log_2_ (1 h in SC_ME_/SC_CSH_ medium) (Figure 8A, red bars; Supplementary Table S8). Unfortunately, these sets of proteins could not be related to one another with GO terms exhibiting a *P*-value equal to or below 0.01. There were, however, four regulatory proteins whose levels changed dramatically: Rio1, which is a serine kinase that participates in cell cycle regulation and rDNA integrity; Snt2, which is a subunit of the Snt2 complex RING finger ubiquitin ligase; Cdc36, which participates in regulating mRNA levels; and Cet1, an RNA 5’ triphosphatase that participates in mRNA 5’ capping.

The levels of 18 proteins in wild-type cells increased by a log_2_ values >5 (green bars) and four decreased by a log_2_ values smaller than −5 (red bars) 6 h after being transferred to the SC_ME_ medium (Figure 8B; Supplementary Table S9). Four of these proteins were the same ones whose levels increased after 1 h of incubation in SC_ME_ medium, Gim4, Snt2, Apa2, and YJL133C-A (asterisks in Figure 8, A and B). In contrast to expectation, the only NCR-sensitive protein was Ecm15 whose function is unknown. The most striking characteristic of the relatively large group of proteins in Figure 8B was that they were not significantly associated with any GO process and the only significant GO function was isocitrate dehydrogenase activity for the mitochondrial Idh1 and Idh2 proteins.

However, four of the 43 proteins in Figure 8B were loosely associated with carbohydrate metabolism/glycolysis: Gdb1, glycogen debranching enzyme required for glycogen degradation; Sol4, 6-phosphogluconolactonase which increases in response to DNA replication stress; Gcy1, glycerol dehydrogenase which is involved in glycerol catabolism under microaerobic conditions; and Tdh2, glyceraldehyde-3-phosphate dehydrogenase that participates in glycolysis and gluconeogenesis. Also of potential significance, Tip41, the Tap42 Interacting Protein, increased by a log_2_ value >7 (Figure 8B). Tip41 is a negative regulator of TorC1 that activates the PP2A-like phosphatase Sit4 by competing with its binding to Tap42 (Jacinto *et al.* 2001). It is interesting that even though there were 43 proteins whose levels changed by log_2_ values of >1 in Figure 8B, they did not appear to be functionally related in a GO process term analysis.

### Major protein targets altered only in the *whi2Δ*

**Figure 9:**
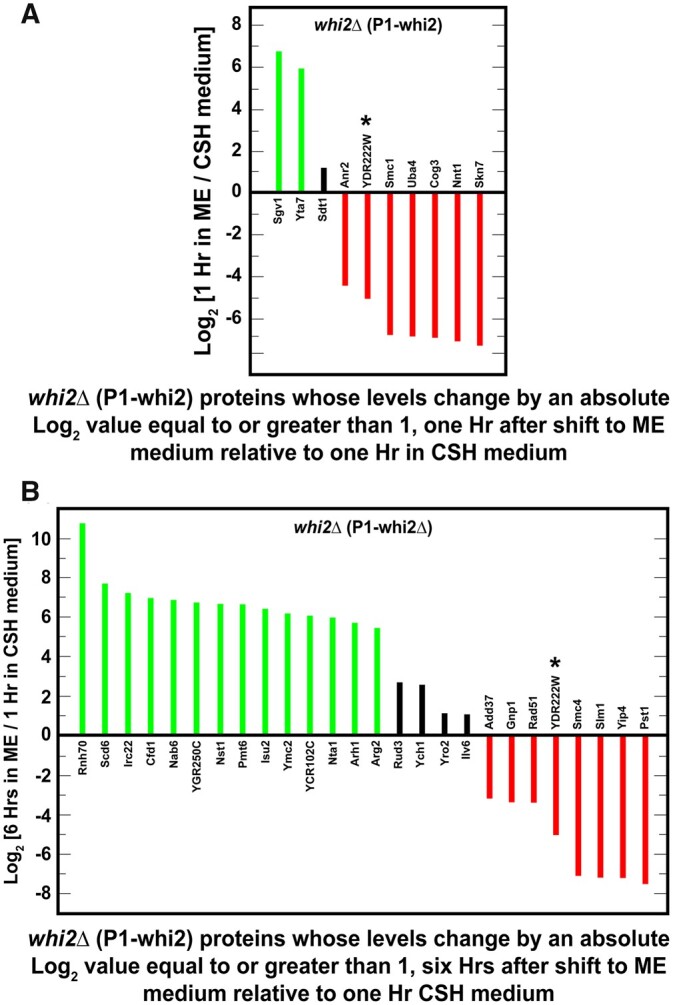
Proteins whose levels changed by absolute log_2_ values ≥1 (*i.e.*, equal to or greater than twofold) in *whi2*Δ (P1-whi2) but not wild-type (P1) cells. Cells were cultured for 1 h (A) or 6 h (B) in SC_ME_ medium and the results compared with those obtained after cells were cultured for 1 h in SC_CSH_ medium. Prior to the beginning of the experiment, cells were pregrown overnight in SC_CSH_ medium to an *A*_600 nm_ = 0.35. SGD GO process analyses of the genes in (A) and (B) did not yield any significant results with *P*-value <0.01.

When a *whi2Δ* strain was transferred from SC_CSH_ to the poorer SC_ME_ medium for 1 h, the levels of 10 proteins changed by absolute log_2_ values >1 (Figure 9A; Supplementary Table S10). Of these, two proteins increased by log_2_ values >5 (green bars), thereby yielding positive values for log_2_ (1 h in SC_ME_/SC_CSH_ medium). In contrast, seven proteins decreased by log_2_ values <−4 to −7× (red bars). Again, there were no GO process terms with *P*-values equal to or below 0.01 associated with this group of proteins.

Extending the time in the SC_ME_ medium to 6 h resulted in a greater number of proteins being increased (14, green bars) or decreased (8, red bars) by an absolute log_2_ value >5 (Figure 9B; Supplementary Table S11). Again, however, they were not significantly associated with any GO process terms. Note that despite these large changes, only one of these proteins were represented among proteins whose levels changed both after 1 and 6 h (Figure 9, A and B). Overall, it was surprising that of the more than 90 wild-type and *whi2Δ* proteins whose levels changed uniquely in only the wild type or the *whi2Δ*, we were unable to associate them with a GO process term.

### Proteins whose levels differed when comparing *whi2Δ vs* wild-type SC_CSH_-grown cells

**Figure 10:**
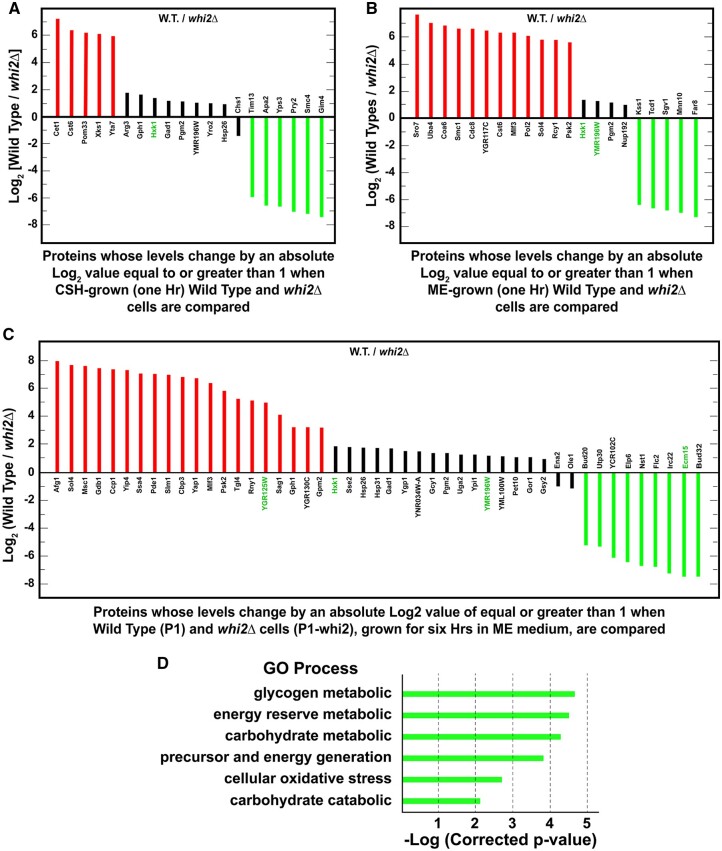
Proteins whose levels differed by absolute log_2_ values ≥1 (twofold), in a *whi2*Δ relative to wildtype cells grown under the same condition. All cultures were pregrown overnight in SC_CSH_ medium to an *A*_600 nm_ = 0.35. (A) Cells were cultured in SC_CSH_ medium. (B) Cells were transferred from SC_CSH_ to SC_ME_ medium and grown for 1 h before being assayed. (C) Cells were transferred from SC_CSH_ to SC_ME_ medium and grown for 6 h prior to assay. Known or potentially NCR-sensitive genes are in green text. (D) GO process analysis for the genes identified in (C). GO process analyses for genes in (A) and (B) were negative when a *P*-value of 0.01 was used.

To approach the proteomic data from a third vantage point, we identified proteins whose levels were markedly different when evaluated in wild-type *vswhi2Δ* cells. In amino acid rich SC_CSH_ medium, six proteins increased by log_2_ values >5 in a *whi2Δ* compared with wild type, thereby yielding log_2_ (Wild Type/*whi2Δ*) that were negative (Figure 10A, green bars; Supplementary Table S12). They were Tim13, a mitochondrial import translocase associated with inserting hydrophobic proteins into the mitochondrial inner membrane; Apa2, diadenosine tetraphosphate phosphorylase involved in the catabolism of nucleosidyl tetraphosphates; Yps3, aspartate yapsin-family protease associated with cell wall growth and maintenance; Pry2 a sterol binding protein associated with the export of fatty acids; Smc4, a subunit of chromosome condensin complex acting during mitosis and meiosis; and Gim 4, prefoldin subunit 2 which binds to cytosolic chaperonin and transfers target proteins to it.

Thirteen proteins decreased by log_2_ values <−1 with five decreasing by log_2_ values <−5 in a SC_CSH_-grown *whi2Δ* compared with wild type, thereby yielding positive values for log_2_ (Wild Type/*whi2Δ*) (Figure 10A, red bars Supplementary Table S12). The five proteins most decreased in the *whi2Δ* were Cet1, which participates in mRNA 5’ capping; Cst6, a basic leucine zipper transcription factor that participates in the stress response regulatory network; and Pom33 a nucleoporin; Xks1, xylulose kinase; and Yta7, a chromatin-binding ATPase regulating histone gene expression. Although these five proteins were insufficiently related to generate a positive GO correlation, Cet1, Cst6, Pom33, and Yta7 are loosely related to RNA metabolism. Interestingly, the fifth member of this group encodes xylulose kinase the rate limiting step in xylulose metabolism.

Four of the 13 proteins identified were highly enriched for the GO process, carbohydrate catabolism −log(*corrected *P*-value) = 2.16; *P*-value = 0.007: Xks1 (xylulokinase), Gph1 (glycogen phosphorylase), Hxk1 (Hexokinase 1), and Pgm2 (phosphoglucomutase) (Supplementary Table S13). On the other hand, only two of the 20 proteins whose levels increased or decreased in the *whi2Δ* relative to wild type were associated with amino acid metabolism, Gad1 catalyzing the decarboxylation of glutamate that participates in a response to oxidative stress and Arg3 required for the biosynthesis of citrulline and arginine. Changes in the levels of these proteins were moderate (absolute log_2_ values of 1–2) relative to the eleven proteins whose levels changed the most (Figure 10A, red and green bars).

*A corrected *P*-value is the smallest familywise significance level at which a particular comparison will be declared statistically significant as part of the multiple comparison testing.

### Proteins whose levels differed when comparing a *whi2Δ* to wild-type cells were transferred from SC_CSH_ to SC_ME_ medium for 1 h

The lack of Whi2 had a great effect on 21 proteins relative to wild type when cells were transferred from SC_CSH_ to poorer SC_ME_ medium for 1 h (Figure 10B; Supplementary Table S14). Of these 21 proteins, five increased by log_2_ values >6 in the *whi2Δ* thereby yielding negative values for log_2_ (Wild Type/*whi2Δ*) (green bars). They were Far8, which acts in the cell cycle arrest recovery process; Mnn10, a subunit of the Golgi mannosyltransferase complex; Sgv1, a cyclin (Bur2p)-dependent protein kinase functioning in transcription; Tcd1, tRNA threonylcarbamoyladenosine dehydratase required for tRNA base modification and Kss1, an MAPK kinase involved in signal transduction pathways that control filamentous growth and pheromone response.

Twelve proteins decreased by log_2_ values <−5 to −7 and four by log_2_ values of <−1 to −2 when the *whi2*Δ was grown in SC_ME_ for 1 h compared with wild type, thereby yielding positive values for log_2_ (Wild Type/*whi2*Δ) (Figure 10B, red and black bars, respectively; Supplementary Table S14). Three of these proteins exhibiting decreased levels in this condition were highly enriched [−log(*corrected *P*-value) = 2.24] for the GO processes associated with glucose-6-phosphate metabolism, and more specifically the pentose phosphate pathway (Supplementary Table S15). They were Sol4 (the gene encoding Sol4 was isolated as a suppressor of the los1-1 mutation), 6-phosphogluconolactonase 4, converting 6-phosphogluconolactone to 6-phosphogluconic acid required for the oxidative phase of the pentose pathway, which decreased by nearly 6×; Hxk1, hexokinase 1, catalyzing phosphorylation of glucose to yield glucose-6-phosphate, which is highly derepressed when cells are provided with nonglucose carbon sources and is the first step in the conversion of glucose to pentoses; and Pgm2, phosphoglucomutase, catalyzing the interconversion of glucose-6-phosphate and glucose-1-phosphate, G-1-P). It is pertinent that G-1-P is the first unique step in the pentose phosphate pathway that also participates in glycogen and trehalose metabolism. Hxk1 and Pgm2 were decreased only modestly by a log_2_ values between about −1 to −1.4. in the *whi2*Δ after 1 h in SC_ME_ medium. Together, these data suggested that the pentose phosphate pathway was significantly downregulated in a *whi2*Δ.

Seven proteins, whose levels increased or decreased (by an absolute log_2_ value ≥1) relative to wild type after the *whi2*Δ was grown for 1 h SC_ME_ medium, were highly enriched [-log(*corrected *P*-value) = 2.76] for the GO function, transfer of phosphorus groups (Figure 10B; Supplementary Tables S14 and S16): Hxk1, Hexokinase 1; Pol2, the catalytic subunit of DNA polymerase ε; Kss1, the MAPK that controls filamentous growth; Cdc8, a nucleoside monophosphate—nucleoside diphosphate kinase; Psk2, a serine/threonine protein kinase that coordinates the regulation of sugar flux and translation; Sgv1, the cyclin-dependent protein kinase component of the BUR complex, phosphorylates the C-terminal domains of RNA polymerase II and elongation factor Spt5-Sgv1; Uba4, an E1-like protein that acts in the thiolation of the wobble base of tRNAs and Sro7, whose loss prevented filamentation and invasive growth in Σ−1278 b strains. All but Hxk1 increased or decreased by an absolute log_2_ values >5.

### Proteins whose levels differed between a *whi2*Δ and wild type downshifted from SC_CSH_ to SC_ME_ medium for 6 h

After 6 h of growth in the poorer SC_ME_ medium, 20 of the 47 proteins decreased by log_2_ values <3 in the *whi2*Δ, thereby yielding positive values for log_2_(Wild Type/*whi2*Δ) (Figure 10C, red bars; Supplementary Table S17). Eleven of these proteins were highly enriched for the GO process, carbohydrate, and energy metabolism (Figure 10D; Supplementary Table S18). The four proteins most affected, decreased by log_2_ values between −3 and −8: Gph1, a glycogen phosphorylase; Psk2 serine/threonine kinase; Gdb1, a glycogen debranching enzyme; and Sol4, the phosphogluconolactonase required for the oxidative branch of the pentose pathway. The others decreased by only log_2_ values of <−1 to −2 (Figure 10C, black bars up to Gsy2).

There were also nine proteins whose levels increased by log_2_ values >4–7 in the *whi2*Δ thereby yielding negative values for log_2_ (Wild Type/*whi2*Δ) (Figure 10C green bars; Supplementary Table S17). However, their functions were not easily related to one another. They were Bud20, a zinc finger protein required for ribosome assembly; Utp30, a subunit of U3-containing 90S preribosome complex; YCR102C, a putative quinone oxidoreductase associated with acid stress resistance; Elp6, a RecA-like ATPase Elp456 Elongator subcomplex required for modification of tRNA; Nst1, a protein involved in signal transduction pathways mediating responses through cell wall integrity, high-osmolarity glycerol and pheromone pathways; Flc2, a putative calcium channel involved in calcium release under hypotonic stress, required for uptake of FAD into endoplasmic reticulum and involved in cell wall maintenance; Irc22, a protein of unknown function that may localize to the ER; Ecm15, a protein that may be associated with cell wall biogenesis and Bud32, a Protein kinase that is a component of the EKC/KEOPS complex which is required for tRNA modification and telomeric recombination.

Only four of the 47 proteins in Figure 10C were related to NCR-sensitive regulation (green text). One, YMR125W, an uncharacterized vacuolar membrane protein decreased by a log_2_ value of nearly −5 in a *whi2*Δ. Also decreased, but only by log_2_ values of −1 to −3, were the putatively NCR-sensitive proteins, Hxk1 and YML196W. In contrast, the uncharacterized vacuolar membrane protein, Emc15, increased in the *whi2*Δ by a log_2_ value >7.

## Discussion

### Explanation of *DAL80* expression *vs* Dal80-GFP production in SC medium

Present experiments were initiated in response to a paradoxical question, how to explain highly NCR-sensitive *DAL80* expression in nitrogen replete SC medium containing 0.5% ammonium sulfate plus 0.18% (SC_CSH_) or 0.12% (SC_ME_) total amino acids? The major NCR-sensitive, TorC1-responsive transcription activator, Gln3, was not demonstrably responsible for the *DAL80* expression. It remained staunchly sequestered in the cytoplasm in these excess nitrogen conditions in both wild type and a *whi2*Δ. In contrast, Gat1—being more resistant to NCR (Georis *et al.* 2008), autogenously regulated (Coffman *et al.* 1996), and a significant contributor to *DAL80* expression (Cunningham *et al.* 2000)—did modestly enter the nuclei of wild-type and *whi2*Δ cells. Therefore, we suggest that Gat1 likely accounted for the small amount of *DAL80* expression we could demonstrate using qPCR assays as well as the modest effect of a *whi2*Δ on that expression: a decrease of 50% after 6 h in SC_ME_, but only 18% when *DAL80* expression was more fully derepressed in proline medium.

### Whi2 is only a minor regulator of NCR-sensitive protein production

The modest NCR-sensitive *DAL80* expression argued that Whi2 was only a minor regulator of NCR-sensitive protein production. This conclusion was supported by our proteomic data. Only 16 of 125 known or potential NCR-sensitive proteins, or proteins emanating from genes with GATA elements in their promoters exhibited significant changes in our combined analyses. Further, only three of these 16 were associated with catabolic activity and those three participated in amino acid interconversions. In contrast, 32 of the 125 proteins were present in the proteomic data but did not change. By inductive reasoning, if one accepts that the effects of the *whi2*Δ on *DAL80* expression likely derived from the downregulation of TorC1, then one must also conclude that Whi2 only modestly regulates TorC1 after 6 h of growth in SC_ME_ medium. This is consistent with the earlier observation that a low concentration of rapamycin (2.5 ng/ml) was sufficient to suppress overgrowth of *whi2* mutants relative to wild type (Chen *et al.* 2018).

One of the NCR-sensitive proteins that failed to appear in our proteomic data was Mep2, ammonia permease. That may have occurred for purely technical reasons. However, it prompted us to look for evidence of Whi2 controlling MEP gene expression. In doing so, we realized a curious set of observations. Mep2 is activated via its phosphorylation by Npr1, which is upregulated when TorC1 is downregulated (Vandenbol *et al.* 1990; Schmidt *et al.* 1998; Feller *et al.* 2006; Tate *et al.* 2006; Boeckstaens *et al.* 2014). Since Whi2 downregulates TorC1, one would *a priori* expect it to upregulate Npr1 and Mep2 activities. However, Boeckstaens *et al.* (2014) demonstrated Whi2’s binding partners, Psr1/Psr2 dephosphorylate and downregulate Mep2 activity.

### Extended growth in SC_ME_ medium elicits large-scale reorganization of amino acid metabolism

Despite the quite modest Whi2-dependent regulation of NCR-sensitive transcription, dramatic changes were observed to be independent of Whi2 when cells were transferred from SC_CSH_ to SC_ME_ medium. Amino acid biosynthesis dramatically increased. Eighty-one proteins significantly increased and did so more or less equivalently in wild-type and *whi2*Δ cells (Figure 2). In contrast, only 10 proteins decreased. This massive reorganization of amino acid metabolism did not, however, involve all amino acids. The predominant increases occurred for basic arginine, lysine and histidine, aliphatic leucine, isoleucine and methionine, aromatic phenylalanine, tryptophan and tyrosine, and serine-related asparagine and glycine. On the other hand, proteins required to synthesize multiple other amino acids that were omitted completely in the SC_ME_ medium, *i.e.*, cystine, glutamine, and proline, were largely unaffected, which coincides with earlier observations made in a *gcn2*Δ at the level of tRNA charging (Zaborske *et al.* 2010). Together, these observations argued that Whi2 was not a significant regulator of amino acid biosynthesis.

It is worthy of emphasis that the nine amino acids whose biosynthesis increased in this study are the same ones whose concentrations were decreased in SC_ME_ relative to SC_CSH_ medium (Supplementary Table S1). This likely contributes to explaining why multiple amino acids, in addition to leucine, were needed to overcome the effect of a *whi2*Δ (Teng *et al.* 2018). The importance of the correlations we report is that cellular events differed significantly in the different SC formulations and did so over the time of incubation in them. Hence the extent to which these differences are important to future investigations, different formulations of SC media cannot be prudently used interchangeably.

### Gcn2, Whi2, and regulation of TorC1 and amino acid biosynthesis in SC_ME_ medium

The highly increased production of amino acid biosynthetic pathway proteins, we observed in Figure 2 and Supplementary Table S4 prompts us to query the regulatory systems involved. Here, the general amino acid control (GAAC) pathway immediately comes to mind (Hinnebusch 1988, 1993, 2014). In general, amino acid limitation results in decreased charged tRNA levels which in turn activate Gcn2 kinase that inhibits overall protein synthesis and TorC1 activity (Zaborske *et al.* 2009, 2010; Staschke *et al.*, 2010; Yuan *et al.* 2017). The inhibition of TorC1 by both Whi2 and Gcn2 raises an important, unanswered question. Do Gcn2 and Whi2 function in parallel to inhibit TorC1 or alternatively in tandem?

Gcn2 activity also stimulates translation of a select group of mRNAs, including that of *GCN4*, and is required for nuclear Gln3 localization. Gcn4 is a central participant in the activation of many amino acid biosynthetic, nutrient reutilization and some stress-related genes (Hinnebusch and Natarajan 2002; Hinnebusch 1994). This could straightforwardly lead to the conclusion that GAAC control explains the increased amino acid pathway proteins and *DAL80* expression we observed. Explanations of our data, however, are more complicated.

Zaborske *et al.* (2009, 2010) measured the growth and charging profiles of all tRNAs in wild-type and *gcn2*Δ cells cultured in SC_ME_ medium from which each of the 20 amino acids were individually omitted. In wild-type cells, none of the omissions had a significant effect on tRNA charging levels or growth. Remarkably in the *gcn2*Δ, the omission of only tryptophan or arginine gradually affected growth and tRNA charging levels. In the case of tryptophan deficiency, the tRNA charging levels were restored if tyrosine and phenylalanine were omitted along with tryptophan or all three amino acids were present. This result argued that both GAAC and release from metabolite feedback inhibition of aromatic amino acid biosynthesis were necessary to maintain a wild-type response. In the case of arginine, analogous analyses argued that both GAAC and arginine-related metabolites, ornithine, and citrulline influence the capacity for arginine biosynthesis.

Our findings of increased methionine and arginine biosynthetic pathway proteins 1 h after transferring cells to SC_ME_ medium, correlates well with the findings of Zaborske *et al.* (2009, 2010). In the case of methionine, Zaborske *et al.* (2010) observed that omission (or limiting concentrations) of tryptophan, one of the amino acids whose concentration is reduced in SC_ME_ medium, also decreased charged tRNA^MET^. However, by this reasoning, why did we not see the aromatic amino acid biosynthetic pathway proteins increased after 60 min in SC_ME_ medium? We suggest this result derives from the fact that tryptophan, phenylalanine, and tyrosine were all present in the SC_ME_ medium, and that there was still sufficient tryptophan present at 1 h in SC_ME_ medium to mitigate the presence of phenylalanine and tyrosine, the feedback inhibitors of the pathway. However, by 6 h in SC_ME_ medium this was no longer the case and one observes significant increases in aromatic amino acid biosynthetic proteins.

We also observed increased levels of aliphatic branched chain amino acid biosynthetic proteins 1 h after transfer to SC_ME_ medium. Zaborske *et al.* (2010), however, did not see a change in tRNA profiles when these amino acids were omitted. Here, we suggest that the leucine auxotrophy of our strains abrogated or reduced the capacity for aliphatic branched-chain amino acid synthesis. That coupled with transferring cells to SC_ME_ medium, further reducing leucine availability to the growing cells, triggered the results we observed. All of these effects were amplified 6 h after transfer of the cells to SC_ME_ medium as all of the amino acids were being depleted by the increased number of cells assimilating them.

Finally, why did Gcn2 activation, as signaled by increased amino acid biosynthesis, fail to elicit greater nuclear Gln3 localization and increased expression of NCR-sensitive genes? This is likely because the regulation of intracellular Gln3 localization is multifaceted: (i) nuclear Gln3 localization is inhibited by upregulation of TorC1 and downregulation of Gcn2 that occurs in nitrogen replete medium (Tate *et al.* 2017). It is also important to recall that TorC1 and Gcn2 reciprocally regulate one another’s activities (Cherkasova and Hinnebusch 2003; Yuan 2017). As amino acids are lower and depleted by growth in the SC_ME_ medium, Gcn2 activity increases, but overall nitrogen availability both from remaining amino acids and *de novo* assimilation of ammonia remains high. Our Gln3 localization and NCR-sensitive protein production data suggest that TorC1’s negative regulation of Gln3 is stronger than is Gcn2’s positive regulation. Further, wild-type Whi2 activity along with Gcn2 activation after 6 h in SC_ME_ medium still remains insufficient to overcome the negative regulation of Gln3 localization. Hence Gln3 localization remains almost completely cytoplasmic and NCR-sensitive gene expression minimal. (ii) Additionally, intracellular glutamine concentration, which would remain high in ammonia assimilation, elicits rapid Gln3 exit from the nucleus before it can activate NCR-sensitive transcription (Rai *et al.* 2015). These explanations must remain tentative, however, because they do not adequately address the possibility that the downstream effects of TorC1, Gcn2 and Whi2 are likely individually and/or collectively graded, and if so, to what extent(s). That Whi2 so minimally affects NCR-sensitive gene expression in the face of much greater control of Rps6 phosphorylation argues strongly in favor of such graded downstream responses by these global regulators (present work and Chen *et al.* 2018).

Our data also suggest that the relative NCR-insensitivity and autogenous regulation of *GAT1* expression and Gat1 activity likely accounts for the little nitrogen-responsive *DAL80* expression we observed (Georis *et al.* 2008). The evidence, *DAL80* expression increases after 6 h’s relative to 1 h’s growth in SC_ME_ medium. Present data do not, however, answer the question, does the increase derive from increased Gcn2 or decreased TorC1 activities or both? Whi2 modestly influences that expression in SC_ME_ but not SC_CSH_ medium. What is clear is that the increase is small relative to normal NCR-sensitive derepression. The extent of Whi2’s influence on *DAL80* expression appears to be independent of the growth-time and hence amino acid concentration in SC_ME_ medium because *DAL80* expression was similarly lowered (∼50%) at both growth times in *whi2*Δ cells. This may account for the similar amounts of Dal80-GFP observed in the work of Chen *et al.* (2018).

### The major targets of Whi2 were not demonstrably related

The levels of many proteins drastically changed in a strain-specific manner after 1 and 6 h in SC_ME_ medium; 57 in the case of wild-type cells and 35 in the *whi2*Δ (Figures 8B and 9b). There were three outstanding characteristics of the changes we observed during our experiments: (i) a large majority of the changes were dramatic, by absolute log_2_ values >3 and could be speculated to be binary on or off in terms of a protein’s presence. (ii) Whi2 affects processes far more diverse than those expected if its primary function is to negatively regulate TorC1 activity. Further, in most cases it was not possible to obtain a GO analysis result with a *P*-value of <0.01. In the single case where GO data were obtained, *i.e.*, comparing wild type to *whi2*Δ protein levels after 6 h in SC_ME_ medium, the protein relationships center on carbohydrate metabolism, energy generation, and stress responses. (iii) In very few cases did the changed proteins behave coordinately in the 1- and 6-h samples; four in wild type and only one in the *whi2*Δ. These data again emphasize the need for caution in the interpretation of data collected in different, even seemingly highly related media, and different conditions of growth and experimental perturbation. The remarkable dynamics exhibited by strains, as they grow, should be no surprise though at times overlooked.

A further characteristic that clearly distinguished the wild-type and *whi2*Δ strain protein compositions was a difference in the number of regulatory proteins whose levels changed, seven in the case of wild type compared with only three in the *whi2*Δ (Figures 8 and 9; Supplementary Tables S8–S11). In wild-type cells grown 1 h in SC_ME_ medium, three control proteins increased by log_2_ values >6: Rio1, a serine kinase involved in cell cycle regulation and rDNA integrity; Snt2, a ring-finger ubiquitin ligase (E3) that binds with other proteins to the promoters of some stress response genes; and Cdc36 that participates in the transcription and destabilization of mRNAs (Supplementary Table S8). At 6 h in SC_ME_ medium, five regulatory proteins increased by log_2_ values between 4 and 7 in the wild type: Tip41, a Tap42 interacting protein that negatively regulates TorC1 and activates Sit4 phosphatase; Rtk1, a putative protein kinase that is phosphorylated by Cdc28 and increases during DNA replication stress; Ume1, a component of histone deacetylase complexes and negative regulator of meiosis; Atg1, a serine/threonine protein kinase that participates in autophagic vesicle formation; and Snt2 (Supplementary Table S9). Note that only one protein, Snt2, increased at both time points.

In contrast, we identified only two regulatory proteins whose levels change in the *whi2*Δ after 1 h in SC_ME_ medium (Figure 9): Sgv1, a cyclin dependent protein kinase that participates in transcriptional regulation increased by a log_2_ value >6, whereas Skn7, a transcription factor required for induction of heat shock genes responding to oxidative stress, decreased by a log_2_ value <−7 (Supplementary Table S10). At 6 h in SC_ME_ medium, only one regulatory protein increased, Ych1, a Cdc25 family tyrosine phosphatase, by a log_2_ value >2 (Supplementary Table S11). Two additional regulatory proteins decreased by log values >7: Slm1, a phosphoinositide PI4,5P binding protein and TorC1 target that increases response to DNA replication stress; and Yip4, which interacts with Rab GTPases at late Golgi vesicles.

When wild-type and *whi2*Δ proteomes are compared directly (Figure 10), the levels of two serine/threonine protein kinases changed by log_2_ values >5 after 1 h in SC_ME_ medium: Psk2 that regulates sugar flux decreases in the *whi2*Δ whereas, Sgv1, a cyclin-dependent kinase whose loss results in myo-inositol auxotrophy increases to a similar degree (Supplementary Table S14). After 6 h in SC_ME_ medium, four regulatory proteins decreased by log_2_ values <−5 in the *whi2*Δ relative to wild type: Yap1, a transcription factor required for stress tolerance; Ypi1, the regulatory subunit of a Type I protein phosphatase and regulates glycogen metabolism and mitosis. As occurred after 1 h in SC_ME_ medium, Slm1 and Psk2 were also downregulated in a *whi2*Δ (Supplementary Table S17).

### Differences in the wild-type and *whi2*Δ proteomes of liquid cultures compared with colonies on plates

It also was striking how different the effects of a *whi2*Δ were in colonies previously grown in complex respiratory medium on plates compared with the SC liquid cultures described here. 65 proteins increased in *whi2* and *psr1/psr2* colonies relative to wild type (37 by log_2_ values >2.4 and 28 by log_2_ values >0.85) (Maršíková *et al.* 2020; Supplementary Figure S3, A and B). The most significant GO categories observed among these proteins (with log_2_ values >2.4) were those associated with transporter activity or proteins localized to the cell periphery. A smaller difference (log_2_ values >0.85) was observed for metabolic proteins related to alcohol metabolism and polyol synthesis (Maršíková *et al.* 2020; Supplementary Figure S3, A and B). Only one of these 65 proteins identified in colonies was also identified in the current analyses.

In colonies, an additional 31 proteins were identified that were significantly decreased in *whi2* and *psr1*/*psr2* relative to wild type (Maršíková *et al.* 2020; Supplementary Figure S4). Nine of these proteins decreased by log_2_ values <−2.6 to −8.0 and 22 by log_2_ values <−0.8 to −1.9). Significant GO categories (*P*-value log < 0.01) included proteins associated with the cell periphery, extracellular proteins, and proteins involved in cofactor and coenzyme metabolic processes.

Five of the 31 proteins whose levels changed significantly in colonies were also identified in the liquid culture analyses. All five proteins increased in wild type relative to the *whi2*Δ either: (i) after culture in SC_ME_ medium for 6 h (Ygp1, Sag1, Ssa4); (ii) after 1 or 6 h in SC_ME_, or for 1 h in SC_CSH_ (Gad1), or (iii) after 6 h in SC_ME_ and 1 h in SC_CSH_ (Gph1). Two of these proteins are related to the cell response to starvation and stress: cell wall glycoprotein Ygp1, heat shock protein Ssa4, and glutamate decarboxylase Gad1. Three are metabolic proteins: glycogen phosphorylase Gph1 and Gad1, and a third is alpha-agglutinin Sag1p. Expression of the genes for all these proteins is induced with varying intensity during the transition of cells growing on YPD medium to stationary phase (Gasch *et al.* 2000).

It has been previously reported that the Whi2p–Psr1p/Psr2p complex plays a role in the general stress response, with cells defective in this complex being more sensitive to stress (Kaida *et al.* 2002). This stress response is related to the function of the transcriptional regulator Msn2p/Msn4p, which appears to be involved in the regulation of three of the above genes identified in both liquid cultures and colonies on plates (Ygp1p, Ssa4p, and Gph1p).

Overall, a comparison of the proteomic differences between wild type and *whi2* identified in liquid cultures with those identified in colonies cultured on plates showed very little commonality. This result is not surprising since the cultivation conditions were significantly different in both types of experiments—colonies *vs* liquid cultures and complex respiratory medium *vs* fermentative glucose medium with different amino acid additions. Moreover, for colonies, only proteins significantly different from wild type in both *whi2* and *psr1*/*psr2* strains were considered. On the other hand, it is not surprising that there was some agreement between changes observed between colonies and liquid cultures after 6-h of culture in SC_ME_, where a portion of the initial nutrients (including glucose) had been consumed, conditions more similar to those in colonies.

### Conclusions

This work initially investigated unexpected NCR-sensitive *DAL80-GFP* gene expression (Dal80-GFP production) in two nitrogen replete SC media (SC_CSH_ and SC_ME_) that are routinely used interchangeably as reference conditions for yeast physiology investigations. GATA activation factor localization and proteomic data obtained with these media demonstrated: (i) Gln3 is staunchly cytoplasmic irrespective of the SC formulation used and the time wild-type and *whi2*Δ cells are incubated in them, 1 or 6 h. (ii) Gat1, being autogenously regulated and more insensitive to NCR than Gln3, partially localizes to the nucleus accounting for the modest *DAL80* expression observed. (iii) There is massive and equivalent reorientation of amino acid biosynthetic protein production in both wild-type and *whi2*Δ cells transferred from SC_CSH_ to SC_ME_ medium. Whi2 does not play a demonstrable role in regulating this reorientation. However, the amino acid biosyntheses most affected by the transfer are those whose concentrations are diminished in SC_ME_ medium. These observations correlate well with those expected from earlier studies of Gcn2 (GAAC) regulation of amino acid biosynthesis. These results may also contribute to explaining the earlier conclusions that other amino acids in addition to the absolute level of leucine were sensed by wild-type cells and ignored by *whi2*Δ cells. As a result, some or as many as 13 amino acids in addition to low leucine are required to suppress wild-type growth relative to that of *whi2*Δ cells in SC_ME_ medium. (iv) Although loss of Whi2 modestly diminishes *DAL80* expression (twofold), it does not demonstrably regulate overall NCR-sensitive or TorC1-regulated protein production. Loss of Whi2, on the other hand, drastically effects the production of 58 proteins which, with two exceptions—carbohydrate metabolism and oxidative stress, are not related to one another in GO analyses. We suggest that SC_CSH_ and SC_ME_ media cannot be prudently used interchangeably. Further, data from this and earlier works argue that control of TorC1 downstream targets is highly specific and graded. This work also prompts the important question of whether Gcn2 and Whi2 regulate TorC1 in parallel or in tandem. Our data are speculatively consistent with these regulators functioning in tandem.

## Data availability

Following publication, strains and plasmids will be provided upon request, but only for noncommercial purposes. Commercial and commercial-development uses are prohibited. Materials provided may not be transferred to a third party without written consent. This will be done in accordance with NIH guidelines. Publicly available datasets were analyzed in this study. This proteomic data set has accession number PXD0280004 and can be found at http://www.ebi.ac.uk/pride/archive/projects/PXD028004.

Supplementary material is available at *G3* online.

## Supplementary Material

jkab432_Supplementary_Table_S1

jkab432_Supplementary_Table_S2

jkab432_Supplementary_Table_S3

jkab432_Supplementary_Table_S4

jkab432_Supplementary_Table_S5

jkab432_Supplementary_Table_S6

jkab432_Supplementary_Table_S7

jkab432_Supplementary_Table_S8

jkab432_Supplementary_Table_S9

jkab432_Supplementary_Table_S10

jkab432_Supplementary_Table_S11

jkab432_Supplementary_Table_S12

jkab432_Supplementary_Table_S13

jkab432_Supplementary_Table_S14

jkab432_Supplementary_Table_S15_S16

jkab432_Supplementary_Table_S17

jkab432_Supplementary_Table_S18
