# Supplementary material for: Effects of abolishing Whi2 on the proteome and nitrogen catabolite repression-sensitive protein production
Source: G3 (Bethesda). 2021 Dec 17;12(3):jkab432. doi: 10.1093/g3journal/jkab432 (PMC9210300; doi:10.1093/g3journal/jkab432)
Supplement: jkab432_Supplementary_Table_S1 [file jkab432_supplementary_table_s1.docx]

| **Table S-1** |  |  |  |  |  |
| --- | --- | --- | --- | --- | --- |
| **Recipes for Synthetic Complete Medium*** | **mg / L** | **mg / L** |  | **μM / L** | **μM / L** |
| **Medium** | **SC_CSH_** | **SC_ME_** |  | **SC_CSH_** | **SC_ME_** |
| Adenine | 18.4 | 20 |  | 50 | 54 |
| Inositol | 73.4 | 10 |  | 407 | 56 |
| PABA | 73.4 | 0.2 |  | 535 | 1 |
| Uracil | 73.4 | 20 |  | 655 | 178 |
|  |  |  |  |  |  |
| Glucose | 20,000 | 20,000 |  |  |  |
| Ammonium Sulfate | 5,000 | 5,000 |  |  |  |
|  |  |  |  |  |  |
| Alanine | 73.4 | 0 |  | 825 | 0 |
| Arginine | 73.4 | 20 |  | 422 | 115 |
| Asparagine | 73.4 | 0 |  | 489 | 0 |
| Aspartic acid | 73.4 | 100 |  | 552 | 752 |
| Cystine | 73.4 | 0 |  | 418 | 0 |
| Glutamine | 73.4 | 0 |  | 502 | 0 |
| Glutamic acid | 73.4 | 100 |  | 434 | 591 |
| Glycine | 73.4 | 0 |  | 753 | 0 |
| Histidine | 73.4 | 20 |  | 474 | 129 |
| Isoleucine | 73.4 | 30 |  | 569 | 229 |
| Leucine | 367 | 30 |  | 2802 | 229 |
| Lysine | 73.4 | 30 |  | 402 | 164 |
| Methionine | 73.4 | 20 |  | 483 | 134 |
| Phenylalanine | 73.4 | 50 |  | 445 | 303 |
| Proline | 73.4 | 0 |  | 638 | 0 |
| Serine | 73.4 | 400 |  | 599 | 3810 |
| Threonine | 73.4 | 200 |  | 617 | 1681 |
| Tryptophan | 73.4 | 20 |  | 360 | 98 |
| Tyrosine | 73.4 | 30 |  | 406 | 166 |
| Valine | 73.4 | 150 |  | 527 | 128 |
|  |  |  |  |  |  |
| **Total Concentration of Amino Acids** | **1,761.6** | **1,200** |  | **12,718** | **8,529** |
| *0.67% Yeast Nitrogen Base without amino acids |  |  |  |  |  |
| (*final concentrations: 2% glucose + 0.5% ammonium sulfate) |  |  |  |  |  |
