# Supplementary material for: Effects of abolishing Whi2 on the proteome and nitrogen catabolite repression-sensitive protein production
Source: G3 (Bethesda). 2021 Dec 17;12(3):jkab432. doi: 10.1093/g3journal/jkab432 (PMC9210300; doi:10.1093/g3journal/jkab432)
Supplement: jkab432_Supplementary_Table_S2 [file jkab432_supplementary_table_s2.docx]

**Table S-2**

**Wild Type and/or *whi2*Δ proteins whose levels change by a Log_2_ value greater than 1 after one Hr in ME medium compared to those after one Hr in CSH medium**

| Gene | Log_2_  P1  1 Hr ME | Log_2_  P1  1 Hr CSH | Significance | Log_2_  P1 1 Hr ME/  P1 1 Hr CSH | Log_2_  P1-whi2  1 Hr ME | Log_2_  P1-whi2  1 Hr CSH | Significance | Log_2_  P1-whi2 1 Hr ME  / P1-whi2 1 Hr CSH | Function |
| --- | --- | --- | --- | --- | --- | --- | --- | --- | --- |
| ARG1 | 27.94 | 26.62 | 0.000123 | **1.32** | 27.70 | 26.85 | 0.000004 | **0.85** | Argininosuccinate synthase OS |
| ARG3 | 24.55 | 23.24 | 0.020447 | **1.31** | 23.59 | 21.44 | 0.000276 | **2.16** | Ornithine carbamoyltransferase OS |
| CHA1 | 26.62 | 23.65 | 0.000012 | **2.96** | 26.17 | 22.85 | 0.000109 | **3.32** | Catabolic Ser/Thr dehydratase |
| LEU1 | 29.60 | 27.74 | 0.000003 | **1.86** | 29.45 | 27.72 | 0.000002 | **1.74** | 3-isopropylmalate dehydratase OS |
| MET17 | 27.49 | 26.41 | 0.000287 | **1.08** | 27.21 | 26.34 | 0.000111 | **0.87** | Homocysteine/cysteine synthase OS |
| TMT1 | 24.29 | 22.12 | 0.002476 | **2.17** | 23.91 | 22.12 | 0.005352 | **1.79** | Trans-aconitate 3-methyltransferase OS |
| YHR208W | 28.66 | 27.54 | 0.000121 | **1.12** | 28.55 | 27.58 | 0.000008 | **0.98** | Branched-chain-amino-acid aminotransferase, mitochondrial OS |
