# Supplementary material for: Effects of abolishing Whi2 on the proteome and nitrogen catabolite repression-sensitive protein production
Source: G3 (Bethesda). 2021 Dec 17;12(3):jkab432. doi: 10.1093/g3journal/jkab432 (PMC9210300; doi:10.1093/g3journal/jkab432)
Supplement: jkab432_Supplementary_Table_S4 [file jkab432_supplementary_table_s4.docx]

**Table S-4**

**Wild Type and/or whi2Δ proteins whose levels change by an absolute Log_2_ value equal to or greater than 1 after six Hrs in ME medium compared to those after one Hr is CSH medium**

| Gene | Log_2_  P1  6 Hr ME | Log_2_  P1  1 Hr CSH | *Signific. | Log_2_  P1 6 Hr ME/  P1 1 Hr CSH | Log_2_  P1-whi2  6 Hr ME | Log_2_  P1-whi2  1 Hr CSH | *Signific. | Log_2_  P1-whi2 6 Hr ME  / P1-whi2 1 Hr CSH | Protein |  |  |
| --- | --- | --- | --- | --- | --- | --- | --- | --- | --- | --- | --- |
| AAT1 | 22.09 | 20.96 | 0.008907 | **1.13** | 22.15 | 19.28 | 0.010738 | **2.87** | Aspartate aminotransferase, mitochondrial OS | | |
| AAT2 | 28.65 | 27.65 | 0.000019 | **1.00** | 28.64 | 27.71 | 0.000104 | **0.93** | Aspartate aminotransferase, cytoplasmic OS | | |
| ACO2 | 25.38 | 23.96 | 0.000021 | **1.42** | 25.37 | 24.11 | 0.000030 | **1.26** | Homocitrate dehydratase, mitochondrial OS | | |
| ADE12 | 27.37 | 26.34 | 0.000003 | **1.03** | 27.37 | 26.32 | 0.000198 | **1.06** | Adenylosuccinate synthetase OS | | |
| ALD5 | 28.47 | 26.26 | 0.000004 | **2.21** | 28.43 | 26.25 | 0.000005 | **2.17** | Aldehyde dehydrogenase 5, mitochondrial OS | | |
| ARG1 | 30.77 | 26.62 | 0.000001 | **4.15** | 30.62 | 26.85 | 1.45 e-08 | **3.77** | Argininosuccinate synthase OS | | |
| ARG3 | 27.76 | 23.24 | 0.000025 | **4.52** | 27.57 | 21.44 | 0.000002 | **6.14** | Ornithine carbamoyltransferase OS | | |
| ARG4 | 28.89 | 26.14 | 5.89 e-08 | **2.75** | 28.91 | 26.59 | 4.41 e-08 | **2.32** | Argininosuccinate lyase OS | |  |
| ARG5,6 | 28.52 | 25.29 | 1.39 e-08 | **3.23** | 28.30 | 24.94 | 0.000019 | **3.37** | Protein ARG5,6, mitochondrial OS | | |
| ARG7 | 27.74 | 25.07 | 0.000003 | **2.67** | 27.54 | 25.21 | 0.000001 | **2.33** | Arginine biosynthesis bifunctional protein ArgJ, mitochondrial OS | | |
| ARG8 | 28.05 | 24.43 | 0.000002 | **3.62** | 27.85 | 23.94 | 0.000003 | **3.91** | Acetylornithine aminotransferase, mitochondrial OS | | |
| ARO3 | 27.66 | 25.82 | 0.000119 | **1.84** | 27.53 | 25.93 | 0.000043 | **1.61** | Phospho-2-dehydro-3-deoxyheptonate aldolase, phenylalanine-inhibited OS | | |
| ARO4 | 29.11 | 27.48 | 0.000264 | **1.63** | 28.98 | 27.57 | 0.000507 | **1.40** | Phospho-2-dehydro-3-deoxyheptonate aldolase, tyrosine-inhibited OS | | |
| ARO9 | 24.69 | 23.42 | 0.00033 | **1.27** | 24.52 | 23.59 | 0.00020 | **0.93** | Aromatic amino acid aminotransferase 2 | | |
| ASN1 | 29.68 | 28.29 | 0.000006 | **1.38** | 29.80 | 28.40 | 0.000001 | **1.40** | Asparagine synthetase [glutamine-hydrolyzing] 1 OS | | |
| BAT2 | 27.23 | 25.41 | 0.000015 | **1.82** | 26.96 | 25.69 | 0.000070 | **1.27** | Branched-chain-amino-acid aminotransferase, cytosolic OS | | |
| BNA1 | 27.15 | 25.03 | 0.000044 | **2.13** | 26.64 | 25.02 | 0.000410 | **1.62** | 3-hydroxyanthranilate 3,4-dioxygenase OS | | |
| BUD20 | 15.00 | 21.34 | S | **-6.34** | 20.23 | 21.06 | 0.00362 | **-0.82** | Bud site selection protein 20 OS | | |
| CCC1 | 22.91 | 21.98 | 0.001030 | **0.93** | 22.73 | 19.63 | 0.001664 | **3.11** | Protein CCC1 OS |  |  |
| CHA1 | 25.44 | 23.65 | 0.000456 | **1.79** | 25.22 | 22.85 | 0.000286 | **2.37** | Catabolic L-serine/threonine dehydratase OS | | |
| CIT2 | 24.56 | 22.72 | 0.001756 | **1.84** | 24.03 | 22.55 | 0.000352 | **1.48** | Citrate synthase, peroxisomal OS | | |
| CPA1 | 26.29 | 24.27 | 0.000002 | **2.02** | 26.40 | 24.13 | 1.84 e-07 | **2.28** | Carbamoyl-phosphate synthase arginine-specific small chain OS | | |
| CPA2 | 29.91 | 27.94 | 0.000019 | **1.97** | 29.87 | 28.13 | 1.18 e-07 | **1.75** | Carbamoyl-phosphate synthase arginine-specific large chain OS | | |
| DRE2 | 24.93 | 22.68 | 0.000189 | **2.25** | 24.50 | 22.54 | 0.001921 | **1.96** | Fe-S cluster assembly protein DRE2 OS | | |
| ERG11 | 27.66 | 28.91 | 0.00008 | **-1.26** | 28.30 | 19.19 | 0.000151 | **-0.89** | Lanosterol 14-alpha demethylase | | |
| ERV1 | 24.08 | 22.60 | 0.000122 | **1.48** | 23.94 | 22.66 | 0.001741 | **1.28** | Mitochondrial FAD-linked sulfhydryl oxidase ERV1 OS | | |
| FOL2 | 25.32 | 24.21 | 0.000008 | **1.11** | 25.37 | 24.34 | 0.000106 | **1.04** | GTP cyclohydrolase 1 OS | |  |
| GCV1 | 26.57 | 24.40 | 0.000030 | **2.17** | 26.11 | 23.55 | 0.000002 | **2.56** | Aminomethyltransferase, mitochondrial OS | | |
| GCV2 | 25.99 | 24.17 | 0.000386 | **1.82** | 25.67 | 24.07 | 0.004911 | **1.59** | Glycine dehydrogenase (decarboxylating), mitochondrial OS | | |
| GCV3 | 26.08 | 24.68 | 0.000524 | **1.41** | 25.69 | 24.41 | 0.000033 | **1.28** | Glycine cleavage system H protein, mitochondrial OS | | |
| GDH2 | 25.29 | 23.40 | 0.000084 | **1.89** | 25.05 | 23.80 | 0.000134 | **1.24** | NAD-specific glutamate dehydrogenase OS | | |
| GGC1 | 26.54 | 23.94 | 0.000034 | **2.60** | 26.68 | 24.30 | 0.000002 | **2.38** | Mitochondrial GTP/GDP carrier protein 1 OS | | |
| HES1 | 24.58 | 25.72 | 0.000022 | **-1.14** | 25.21 | 26.16 | 0.000442 | **-0.95** | Regulation of ergosterol biosynthesis | | |
| HHO1 | 22.90 | 23.96 | 0.001237 | **-1.06** | 23.40 | 24.23 | 0.013239 | **-0.84** | Histone H1 OS | | |
| HIS1 | 27.52 | 26.05 | 0.000207 | **1.47** | 27.64 | 25.98 | 0.000018 | **1.65** | ATP phosphoribosyltransferase OS | | |
| HIS4 | 29.89 | 26.69 | 3.27 e-07 | **3.20** | 29.80 | 26.98 | 4.32 e-07 | **2.82** | Histidine biosynthesis trifunctional protein OS | | |
| HIS5 | 27.12 | 24.37 | 0.000002 | **2.75** | 27.26 | 24.61 | 0.000002 | **2.65** | Histidinol-phosphate aminotransferase OS | | |
| HIS7 | 28.12 | 26.76 | 0.000008 | **1.37** | 28.23 | 26.89 | 0.000002 | **1.34** | Imidazole glycerol phosphate synthase hisHF OS | | |
| HOM2 | 30.17 | 28.08 | 0.000016 | **2.09** | 30.18 | 28.20 | 0.000007 | **1.99** | Aspartate-semialdehyde dehydrogenase OS | | |
| HOM3 | 27.01 | 25.43 | 0.000001 | **1.58** | 26.89 | 25.59 | 0.000041 | **1.30** | Aspartokinase OS | | |
| HRB1 | 22.89 | 21.48 | 0.002018 | **1.41** | 22.68 | 21.79 | 0.031565 | **0.89** | Poly(A+) RNA-binding protein | | |
| HSP78 | 26.06 | 24.53 | 0.000016 | **1.53** | 25.45 | 24.54 | 0.000233 | **0.91** | Heat shock protein 78 mitochondrial OS | | |
| HXT2 | 22.56 | 24.95 | 0.000153 | **-2.40** | 23.23 | 25.04 | 0.002171 | **-1.81** | High-affinity glucose transporter HXT2 OS | | |
| IDP1 | 28.85 | 27.67 | 0.000095 | **1.19** | 28.58 | 27.63 | 0.000082 | **0.95** | Isocitrate dehydrogenase NADP mitochondrial | | |
| ILV2 | 30.17 | 27.64 | 0.000002 | **2.53** | 29.97 | 27.72 | 9.20 e-09 | **2.25** | Acetolactate synthase catalytic subunit, mitochondrial OS | | |
| ILV3 | 30.62 | 28.39 | 4.94 e-08 | **2.23** | 30.70 | 28.64 | 3.74 e-07 | **2.06** | Dihydroxy-acid dehydratase, mitochondrial OS | | |
| ILV5 | 31.81 | 30.65 | 0.000002 | **1.17** | 31.84 | 30.72 | 1.49 e-07 | **1.12** | Ketol-acid reductoisomerase, mitochondrial OS | | |
| LEU1 | 32.07 | 27.74 | 0.000001 | **4.33** | 31.95 | 27.72 | 2.46 e-08 | **4.23** | 3-isopropylmalate dehydratase OS | | |
| LEU4 | 30.00 | 26.64 | 9.57e-08 | **3.36** | 29.67 | 26.68 | 1.43 e-07 | **2.99** | 2-isopropylmalate synthase OS | | |
| LEU9 | 25.68 | 24.31 | 0.005910 | **1.37** | 25.67 | 24.18 | 0.000044 | **1.49** | 2-isopropylmalate synthase 2, mitochondrial OS | | |
| LYS1 | 27.86 | 25.35 | 0.000001 | **2.51** | 27.66 | 25.58 | 0.000005 | **2.08** | Saccharopine dehydrogenase [NAD(+), L-lysine-forming] OS | | |
| LYS20 | 26.28 | 23.56 | 0.000032 | **2.72** | 26.25 | 23.52 | 0.000012 | **2.73** | Homocitrate synthase, cytosolic isozyme OS | | |
| LYS21 | 29.29 | 27.60 | 4.16e-07 | **1.70** | 29.12 | 27.80 | 0.000001 | **1.32** | Homocitrate synthase, mitochondrial OS | | |
| MAE1 | 27.61 | 25.94 | 0.000001 | **1.67** | 27.45 | 25.63 | 0.000005 | **1.82** | NAD-dependent malic enzyme, mitochondrial OS | | |
| MET13 | 25.47 | 24.15 | 0.000118 | **1.32** | 25.39 | 24.15 | 0.000968 | **1.24** | Methylenetetrahydrofolate reductase 2 OS | | |
| MET17 | 28.80 | 26.41 | 0.000005 | **2.39** | 28.66 | 26.34 | 0.000001 | **2.32** | Homocysteine/cysteine synthase OS | | |
| MET22 | 27.33 | 25.87 | 0.000046 | **1.45** | 27.20 | 25.98 | 0.000006 | **1.22** | 3(2),5-bisphosphate nucleotidase OS | | |
| MET5 | 24.06 | 22.60 | 0.007093 | **1.46** | 23.23 | 22.08 | 0.043333 | **1.15** | Sulfite reductase [NADPH] subunit beta OS | | |
| MPC2 | 24.37 | 19.50 | 0.000441 | **4.87** | 24.29 | 19.57 | 0.000822 | **4.72** | Mitochondrial pyruvate carrier 2 OS | | |
| NCE103 | 22.98 | 24.37 | 0.000221 | **-1.39** | 23.35 | 24.92 | 0.000744 | **-1.57** | Carbonic anhydrase OS | |  |
| OLE1 | 25.96 | 27.53 | 0.000004 | **-1.57** | 27.12 | 28.14 | 0.000519 | **-1.02** | Acyl-CoA desaturase 1 OS | |  |
| PDR12 | 22.11 | 15.00 | S | **7.11** | 21.73 | 15.00 | S | **6.73** | ATP-dependent permease PDR12 OS | | |
| PDR16 | 23.34 | 22.00 | 0.000506 | **1.33** | 23.31 | 22.37 | 0.007553 | **0.95** | Phosphatidylinositol transfer protein | | |
| PDX3 | 27.44 | 26.15 | 0.000020 | **1.29** | 27.47 | 26.12 | 0.000018 | **1.35** | Pyridoxamine 5-phosphate oxidase OS | | |
| PET10 | 22.74 | 20.76 | 0.000103 | **1.98** | 21.65 | 18.74 | 0.006677 | **2.90** | Perilipin | | |
| QDR2 | 22.67 | 15.00 | S | **7.67** | 22.23 | 15.00 | S | **7.23** | Quinidine resistance protein 2 OS | | |
| REE1 | 26.26 | 24.71 | 0.000882 | **1.55** | 26.13 | 24.70 | 0.000053 | **1.43** | Regulation of enolase protein 1 OS | | |
| RIB5 | 27.20 | 24.91 | 0.000020 | **2.29** | 27.19 | 24.87 | 2.12 e-08 | **2.31** | Riboflavin synthase OS | |  |
| SAG1 | 24.92 | 23.17 | 0.000038 | **1.74** | 20.81 | 22.77 | 0.000087 | **-1.95** | Alpha-agglutinin OS |  |  |
| SAM4 | 27.28 | 25.37 | 0.000088 | **1.91** | 27.03 | 25.52 | 0.000044 | **1.51** | Homocysteine S-methyltransferase 2 OS | | |
| SCW4 | 28.24 | 27.00 | 00.3162 | **1.24** | 28.37 | 24.47 | 0.000945 | **0.89** | Probable family 17 glucosidase OS | | |
| SDT1 | 24.39 | 21.32 | 0.004939 | **3.08** | 24.56 | 21.72 | 0.000324 | **2.84** | Suppressor of disruption of TFIIS OS | | |
| SER1 | 29.13 | 27.68 | 0.000002 | **1.45** | 29.07 | 27.81 | 0.000001 | **1.25** | Phosphoserine aminotransferase OS | | |
| SER3 | 27.16 | 26.04 | 0.000104 | **1.13** | 27.02 | 26.16 | 0.000028 | **0.86** | D-3-Phosphoglycerate dehydrogenase 1 OS | | |
| SNO1 | 24.93 | 15.00 | S | **9.93** | 25.17 | 15.00 | S | **10.17** | Pyridoxal 5-phosphate synthase subunit SNO1 OS | | |
| SPE1 | 15.00 | 22.39 | S | **-7.39** | 15.00 | 22.16 | S | **-7.16** | Ornithine decarboxylase OS | |  |
| SRY1 | 24.56 | 20.61 | 0.000015 | **3.95** | 24.65 | 18.89 | 0.000047 | **5.76** | L-threo-3-hydroxyaspartate ammonia-lyase OS | | |
| TMT1 | 27.36 | 22.12 | 0.000083 | **5.24** | 27.39 | 22.12 | 0.000040 | **5.27** | Trans-aconitate 3-methyltransferase OS | | |
| TRP4 | 25.82 | 23.63 | 0.000004 | **2.19** | 25.72 | 23.78 | 0.000552 | **1.94** | Anthranilate phosphoribosyltransferase OS | | |
| YAT2 | 22.85 | 15.00 | S | **7.85** | 22.64 | 15.00 | S | **7.64** | Carnitine O-acetyltransferase YAT2 OS | | |
| YBR145W | 26.03 | 16.75 | S | **9.27** | 26.71 | 20.70 | 0.000011 | **6.01** | Alcohol dehydrogenase 5 OS | | |
| YBR191W | 24.39 | 26.17 | 0.023811 | **-1.78** | 25.00 | 25.92 | 0.030905 | **-0.92** | 60S Ribosomal protein L21A | | |
| YGL117W | 23.63 | 15.00 | S | **8.63** | 23.58 | 15.00 | S | **8.58** | Uncharacterized protein YGL117W OS | | |
| YHI9 | 26.50 | 22.19 | 0.000534 | **4.31** | 26.70 | 22.93 | 0.000258 | **3.78** | Uncharacterized isomerase YHI9 OS | | |
| YHR208W/  BAT1 | 30.75 | 27.54 | 2.43 e-07 | **3.21** | 30.65 | 27.58 | 1.17e-07 | **3.07** | Branched-chain-amino-acid aminotransferase, mitochondrial OS | | |
| YJR111C | 23.91 | 21.26 | 0.000022 | **2.65** | 23.91 | 21.58 | 0.000016 | **2.32** | Uncharacterized protein YJR111C OS | | |
| YMC1 | 24.20 | 22.82 | 0.000057 | **1.38** | 24.00 | 22.93 | 0.000215 | **1.06** | Mitochondrial glycine transporter YMC1 OS | | |
| YNL129W | 23.46 | 21.88 | 0.000088 | **1.58** | 23.18 | 22.03 | 0.028789 | **1.15** | Nicotinamide riboside kinase OS | | |
| YPI1 | 23.28 | 19.52 | 0.006704 | **3.76** | 22.03 | 19.23 | 0.004189 | **2.80** | Type 1 phosphatases regulator YPI1 OS | | |
| YPL272C | 17.11 | 23.68 | S | **-6.57** | 22.05 | 23.48 | 0.000205 | **-1.42** | Uncharacterized protein | | |
| YSA1 | 26.35 | 25.09 | 0.000001 | **1.26** | 25.80 | 24.97 | 0.01732 | **0.82** | ADP-ribose pyrophosphatase OS | | |
| ZTA1 | 24.53 | 23.38 | 0.001807 | **1.16** | 24.08 | 23.18 | 0.000179 | **0.90** | Probable quinone oxidoreductase OS | | |

***Significance**
