# Supplementary material for: Effects of abolishing Whi2 on the proteome and nitrogen catabolite repression-sensitive protein production
Source: G3 (Bethesda). 2021 Dec 17;12(3):jkab432. doi: 10.1093/g3journal/jkab432 (PMC9210300; doi:10.1093/g3journal/jkab432)
Supplement: jkab432_Supplementary_Table_S8 [file jkab432_supplementary_table_s8.docx]

**Table S-8**

**Wild Type proteins whose levels change by an absolute Log_2_ value greater than 1, one Hr after shift to ME medium relative to CSH medium**

| Gene | Log_2_ P1  1 Hr ME | Log_2_ P1  1 Hr CSH | Significance | Log_2_  P1 ME/ P1 CSH | Function (SGD) |
| --- | --- | --- | --- | --- | --- |
| GIM4 | 22.41 | <15 | S | **7.40** | Prefoldin subunit 2 OS |
| RIO1 | 22.36 | <15 | S | **7.36** | Serine/threonine-protein kinase RIO1 OS |
| SNT2 | 21.85 | <15 | S | **6.85** | E3 ubiquitin-protein ligase SNT2 OS |
| SNZ1 | 21.84 | <15 | S | **6.84** | Pyridoxal 5-phosphate synthase subunit SNZ1 OS |
| BDF1 | 21.77 | <15 | S | **6.77** | Bromodomain-containing factor 1 OS |
| APA2 | 21.74 | <15 | S | **6.74** | Diadenosine 5,5-P1,P4-tetraphosphate phosphorylase 2 |
| ISU2 | 21.29 | <15 | S | **6.29** | Iron sulfur cluster assembly protein 2, mitochondrial OS |
| TSR2 | 21.27 | <15 | S | **6.27** | Pre-rRNA-processing protein TSR2 OS |
| CDC36 | 21.06 | <15 | S | **6.06** | General negative regulator of transcription subunit 2 OS |
| IVY1 | 21.06 | <15 | S | **6.06** | Protein IVY1 OS |
| YJL133C-A | 21.02 | <15 | S | **6.02** | Uncharacterized protein YJL133C-A OS |
| RCY1 | 20.77 | <15 | S | **5.77** | Recyclin-1 OS |
| MPC2 | 22.57 | 19.50 | 0.016819 | **3.07** | Subunit of mitochondrial pyruvate carrier |
| DRE2 | 23.88 | 22.68 | 0.015996 | **1.20** | Component of cytosolic Fe-S assembly machinery |
| GDH2 | 24.50 | 23.40 | 0.001594 | **1.10** | NAD-specific glutamate dehydrogenase OS |
| PDR16 | 23.05 | 22.01 | 0.003200 | **1.05** | Phosphatidylinositol transfer protein PDR16 OS |
| MGR1 | <15 | 21.64 | S | **-6.64** | Mitochondrial inner membrane i-AAA protease supercomplex subunit MGR1 OS |
| CET1 | <15 | 22.24 | S | **-7.24** | mRNA-capping enzyme subunit beta OS |
