# Supplementary material for: Effects of abolishing Whi2 on the proteome and nitrogen catabolite repression-sensitive protein production
Source: G3 (Bethesda). 2021 Dec 17;12(3):jkab432. doi: 10.1093/g3journal/jkab432 (PMC9210300; doi:10.1093/g3journal/jkab432)
Supplement: jkab432_Supplementary_Table_S9 [file jkab432_supplementary_table_s9.docx]

**Table S-9**

**Wild Type, P1, proteins whose levels change by an absolute Log_2_ equal to or greater than 1, six Hrs after shift to ME medium relative to CSH medium**

| Gene | Log_2_ P1  6 Hr ME | Log_2_ P1  1 Hr CSH | Significance | Log_2_  P1 ME/P1 CSH | Function (SGD) |  |
| --- | --- | --- | --- | --- | --- | --- |
| RTK1 | 22.51 | <15.00 | S | **7.51** | Probable serine/threonine-protein kinase RTK1 OS | |
| GDB1 | 22.49 | <15.00 | S | **7.49** | Glycogen debranching enzyme OS | |
| CCP1 | 22.41 | <15.00 | S | **7.41** | Cytochrome c peroxidase, mitochondrial OS | |
| GIM4 | 22.31 | <15.00 | S | **7.31** | Prefoldin subunit 2 OS | |
| TIP41 | 22.31 | <15.00 | S | **7.31** | Type 2A phosphatase activator TIP41 OS | |
| VCX1 | 22.22 | <15.00 | S | **7.22** | Vacuolar calcium ion transporter OS | |
| ARF3 | 22.21 | <15.00 | S | **7.21** | ADP-ribosylation factor 3 OS | |
| FAT1 | 22.21 | <15.00 | S | **7.21** | Very long-chain fatty acid transport protein OS | |
| UME1 | 22.20 | <15.00 | S | **7.20** | Transcriptional regulatory protein UME1 OS | |
| APA2 | 22.18 | <15.00 | S | **7.18** | Diadenosine 5,5-P1,P4-tetraphosphate phosphorylase 2 OS | |
| UBX2 | 22.16 | <15.00 | S | **7.16** | UBX domain-containing protein 2 OS | |
| SSA4 | 22.11 | <15.00 | S | **7.11** | Heat shock protein SSA4 OS | |
| PDE1 | 22.08 | <15.00 | S | **7.08** | 3,5-cyclic-nucleotide phosphodiesterase 1 OS | |
| CBP3 | 21.86 | <15.00 | S | **6.86** | Protein CBP3, mitochondrial OS | |
| SNT2 | 21.62 | <15.00 | S | **6.62** | E3 ubiquitin-protein ligase SNT2 OS | |
| TRP1 | 21.56 | <15.00 | S | **6.56** | N-(5-phosphoribosyl)anthranilate isomerase OS | |
| YJL133C-A | 21.31 | <15.00 | S | **6.31** | Uncharacterized protein YJL133C-A OS | |
| YME2 | 20.68 | <15.00 | S | **5.68** | Mitochondrial escape protein 2 OS | |
| ATG1 | 19.22 | <15.00 | S | **4.22** | Serine/threonine-protein kinase ATG1 OS | |
| SOL4 | 22.72 | 18.88 | 0.000584 | **3.84** | 6-phosphogluconolactonase 4 OS | |
| UGA2 | 23.32 | 19.66 | 0.001239 | **3.66** | Succinate-semialdehyde dehydrogenase [NADP(+)] OS | |
| ARO10 | 22.75 | 19.51 | 0.008454 | **3.24** | Phenylpyruvate decarboxylase | |
| GOR1 | 23.23 | 20.13 | 0.017201 | **3.10** | Glyoxylate reductase 1 | |
| HSP31 | 22.81 | 20.64 | 0.000290 | **2.16** | Glutathione-independent glyoxalase HSP31 OS | |
| TMA10 | 24.06 | 22.51 | 0.000148 | **1.55** | Translation machinery-associated protein 10 OS | |
| GCY1 | 26.45 | 25.16 | 0.000084 | **1.30** | Glycerol 2-dehydrogenase (NADP(+)) OS | |
| YLR179C | 28.27 | 27.00 | 0.000086 | **1.28** | Uncharacterized protein YLR179C OS | |
| AIM17 | 26.83 | 25.65 | 0.000119 | **1.18** | Probable oxidoreductase AIM17 OS | |
| IDH1 | 26.03 | 24.87 | 0.000318 | **1.16** | Isocitrate dehydrogenase [NAD] subunit 1, mitochondrial OS | |
| MTD1 | 23.77 | 22.75 | 0.001979 | **1.02** | Methylenetetrahydrofolate dehydrogenase [NAD(+)] OS | |
| IDH2 | 25.81 | 24.80 | 0.000037 | **1.01** | Isocitrate dehydrogenase [NAD] subunit 2, mitochondrial OS | |
| YJL016W | 21.01 | 22.13 | 0.018378 | **-1.12** | Uncharacterized | |
| ENA2 | 21.95 | 23.18 | 0.001429 | **-1.23** | Sodium transport ATPase 2 OS | |
| TDH2 | 29.53 | 30.81 | 0.002848 | **-1.28** | Glyceraldehyde-3-phosphate dehydrogenase 2 OS | |
| ERP1 | 24.05 | 25.34 | 0.001861 | **-1.28** | Involved in ER to Golgi transport and misfolded quality control | |
| YDR365W-B | 21.94 | 23.39 | 0.018549 | **-1.45** | Transposon Ty1-PL Gag polyprotein | |
| YML039W | 22.48 | 24.05 | 0.050001 | **-1.57** | Transposon Ty-ML2 Gag-Pol polyprotein | |
| ECM32 | 19.46 | 22.27 | 0.003823 | **-2.81** | Putative ATP-dependent RNA helicase ECM32 OS | |
| ALB1 | 20.12 | 23.38 | 0.032157 | **-3.26** | Shuttling pre-60S factor | |
| YBL028C | <15.00 | 20.70 | S | **-5.70** | Predicted to be associated with ribosome biogenesis | |
| ERD2 | <15.00 | 21.99 | S | **-6.99** | ER lumen protein-retaining receptor OS | |
| ECM15 | <15.00 | 22.55 | S | **-7.55** | Unknown function | |
| YLR256W | <15.00 | 22.89 | S | **-7.89** | Heme-responsive zinc finger transcription factor HAP1 OS | |
