# Supplementary material for: Effects of abolishing Whi2 on the proteome and nitrogen catabolite repression-sensitive protein production
Source: G3 (Bethesda). 2021 Dec 17;12(3):jkab432. doi: 10.1093/g3journal/jkab432 (PMC9210300; doi:10.1093/g3journal/jkab432)
Supplement: jkab432_Supplementary_Table_S10 [file jkab432_supplementary_table_s10.docx]

**Table S-10**

***whi2*Δ (P1-whi2) proteins whose levels change by an absolute Log_2_ value equal to or greater than 1, one Hr after shift to ME medium relative to one Hr in CSH medium**

| Gene | Log_2_  P1-whi2  1 Hr ME | Log_2_  P1-whi2  1 Hr CSH | Significance | Log_2_  whi2 ME/whi2 CSH | Function (SGD) |
| --- | --- | --- | --- | --- | --- |
| SGV1 | 21.74 | <15 | S | **6.74** | Serine/threonine-protein kinase BUR1 OS |
| YTA7 | 20.93 | <15 | S | **5.93** | Tat-binding homolog 7 OS |
| SDT1 | 22.92 | 21.72 | 0.008419 | **1.20** | Suppressor of disruption of TFIIS OS |
| ANR2 | <15 | 19.40 | S | **-4.40** | Uncharacterized protein ANR2 OS |
| YDR222W | <15 | 20.04 | S | **-5.04** | SVF1-like protein YDR222W OS |
| SMC1 | <15 | 21.76 | S | **-6.76** | Structural mainte15ce of chromosomes protein 1 OS |
| UBA4 | <15 | 21.87 | S | **-6.81** | Adenylyltransferase and sulfurtransferase UBA4 OS |
| COG3 | <15 | 21.87 | S | **-6.87** | Conserved oligomeric Golgi complex subunit 3 OS |
| NNT1 | <15 | 22.06 | S | **-7.06** | Protein N-terminal and lysine N-methyltransferase EFM7 OS |
| SKN7 | <15 | 22.27 | S | **-7.27** | Transcription factor SKN7 OS |
