# Supplementary material for: Effects of abolishing Whi2 on the proteome and nitrogen catabolite repression-sensitive protein production
Source: G3 (Bethesda). 2021 Dec 17;12(3):jkab432. doi: 10.1093/g3journal/jkab432 (PMC9210300; doi:10.1093/g3journal/jkab432)
Supplement: jkab432_Supplementary_Table_S11 [file jkab432_supplementary_table_s11.docx]

**Table S-11**

***whi2*Δ (P1-whi2) proteins whose levels change by an absolute Log_2_ value equal to or greater than 1, six Hrs after shift to ME medium relative to one Hr in CSH medium**

| Gene | Log_2_  P1-whi2  6 Hrs ME | Log_2_  P1-whi2  1 Hr CSH | Significance | Log_2_  whi2 ME/whi2 CSH | Function (SGD) |
| --- | --- | --- | --- | --- | --- |
| RNH70 | 25.78 | <15.00 | S | **10.78** | RNA exonuclease 1 OS |
| SCD6 | 22.73 | <15.00 | S | **7.73** | Protein SCD6 OS |
| IRC22 | 22.24 | <15.00 | S | **7.24** | Increased recombination centers protein 22 OS |
| CFD1 | 21.98 | <15.00 | S | **6.98** | Cytosolic Fe-S cluster assembly factor CFD1 OS |
| NAB6 | 21.89 | <15.00 | S | **6.89** | RNA-binding protein NAB6 OS |
| YGR250C | 21.75 | <15.00 | S | **6.75** | Uncharacterized RNA-binding protein YGR250C OS |
| NST1 | 21.70 | <15.00 | S | **6.70** | Stress response protein NST1 OS |
| PMT6 | 21.67 | <15.00 | S | **6.67** | Dolichyl-phosphate-mannose--protein mannosyltransferase 6 OS |
| ISU2 | 21.45 | <15.00 | S | **6.45** | Iron sulfur cluster assembly protein 2, mitochondrial OS |
| YMC2 | 21.18 | <15.00 | S | **6.18** | Carrier protein YMC2, mitochondrial OS |
| YCR102C | 21.12 | <15.00 | S | **6.12** | Uncharacterized protein YLR460C OS;Uncharacterized protein YCR102C OS |
| NTA1 | 21.02 | <15.00 | S | **6.02** | Protein N-terminal amidase OS |
| ARH1 | 20.73 | <15.00 | S | **5.73** | Probable NADPH:adrenodoxin oxidoreductase, mitochondrial OS |
| ARG2 | 20.44 | <15.00 | S | **5.44** | Amino-acid acetyltransferase, mitochondrial OS |
| RUD3 | 21.88 | 19.16 | 0.00548 | **2.72** | GRIP domain-containing protein RUD3 OS |
| YCH1 | 21.76 | 19.14 | 0.011709 | **2.62** | Cdc25-Like phosphatase |
| YRO2 | 26.46 | 25.30 | 0.00234 | **1.15** | Protein YRO2 OS |
| ILV6 | 27.84 | 26.80 | 0.000048 | **1.04** | Acetolactate synthase small subunit, mitochondrial OS |
| ADD37 | 19.50 | 22.67 | 0.023733 | **-3.17** | Alpha1-proteinase inhibitor |
| GNP1 | 20.03 | 23.40 | 0.009001 | **-3.37** | High-affinity glutamine permease OS |
| RAD51 | 21.02 | 24.69 | 0.019320 | **-3.66** | DNA repair protein |
| YDR222W | <15.00 | 20.04 | S | **-5.04** | SVF1-like protein YDR222W OS |
| SMC4 | <15.00 | 22.10 | S | **-7.10** | Structural maintenance of chromosomes protein 4 OS |
| SLM1 | <15.00 | 22.19 | S | **-7.19** | Phosphatidylinositol 4,5-bisphosphate-binding protein SLM1 OS |
| YIP4 | <15.00 | 22.21 | S | **-7.21** | Interacts with Rab GTPases in late Golgi |
| PST1 | <15.00 | 22.51 | S | **-7.51** | Cell wall mannoprotein PST1 OS |
