# Supplementary material for: Effects of abolishing Whi2 on the proteome and nitrogen catabolite repression-sensitive protein production
Source: G3 (Bethesda). 2021 Dec 17;12(3):jkab432. doi: 10.1093/g3journal/jkab432 (PMC9210300; doi:10.1093/g3journal/jkab432)
Supplement: jkab432_Supplementary_Table_S12 [file jkab432_supplementary_table_s12.docx]

**Table S-12**

**Proteins whose levels change by an absolute Log_2_ value equal to or greater than 1 when CSH-grown Wild Type (P1) and *whi2*Δ (P1-whi2) cells are compared**

| Gene | Log_2_  P1 | Log_2_  P1-whi2 | Significance | Log_2_  P1/P1-whi2 | Function (SGD) |
| --- | --- | --- | --- | --- | --- |
| CET1 | 22.24 | <15.00 | S | **7.24** | mRNA-capping enzyme subunit beta OS |
| CST6 | 21.40 | <15.00 | S | **6.40** | ATF/CREB activator 2 OS |
| POM33 | 21.22 | <15.00 | S | **6.22** | Pore membrane protein of 33 kDa OS |
| XKS1 | 21.13 | <15.00 | S | **6.13** | Xylulose kinase OS |
| YTA7 | 20.97 | <15.00 | S | **5.97** | Tat-binding homolog 7 OS |
| ARG3 | 23.24 | 21.44 | 0.002083 | **1.80** | Ornithine carbamoyltransferase OS |
| GPH1 | 25.02 | 23.33 | 0.006228 | **1.69** | Glycogen phosphorylase OS |
| HXK1 | 28.65 | 27.23 | 0.000234 | **1.42** | Hexokinase-1 OS |
| GAD1 | 23.50 | 22.29 | 0.000234 | **1.21** | Glutamate decarboxylase OS |
| PGM2 | 27.69 | 26.53 | 0.000163 | **1.17** | Phosphoglucomutase 2 OS |
| YMR196W | 24.04 | 22.98 | 0.013354 | **1.05** | Uncharacterized protein |
| YRO2 | 26.33 | 25.30 | 0.006744 | **1.03** | Putative role in response to acid stress |
| HSP26 | 26.61 | 25.61 | 0.029773 | **1.00** | Heat shock protein 26 |
| CHS1 | 21.68 | 23.07 | 0.030384 | **1.39** | Catabolic L-serine/threonine dehydratase OS |
| TIM13 | <15.00 | 20.86 | S | **-5.86** | Mitochondrial import inner membrane translocase subunit |
| APA2 | <15.00 | 21.49 | S | **-6.49** | Diadenosine 5,5-P1,P4-tetraphosphate phosphorylase 2 OS |
| YPS3 | <15.00 | 21.56 | S | **-6.56** | Aspartic proteinase yapsin-3 OS |
| PRY2 | <15.00 | 21.96 | S | **-6.96** | Sterol binding protein involved in export |
| SMC4 | <15.00 | 22.10 | S | **-7.10** | Structural maintenance of chromosomes protein 4 OS |
| GIM4 | <15.00 | 22.33 | S | **-7.33** | Prefoldin subunit 2 OS |
