# Supplementary material for: Effects of abolishing Whi2 on the proteome and nitrogen catabolite repression-sensitive protein production
Source: G3 (Bethesda). 2021 Dec 17;12(3):jkab432. doi: 10.1093/g3journal/jkab432 (PMC9210300; doi:10.1093/g3journal/jkab432)
Supplement: jkab432_Supplementary_Table_S14 [file jkab432_supplementary_table_s14.docx]

**Table S-14**

**Proteins whose levels change by an absolute Log_2_ value equal to or greater than 1 when Wild Type (P1) and *whi2*Δ (P1-whi2) cells, grown for one Hr in ME medium, are compared**

| Gene | Log_2_ P1  1 Hr ME | Log_2_  P1-whi2  1 Hr ME | Significance | Log_2_  P1/P1-whi2 | Function (SGD) |
| --- | --- | --- | --- | --- | --- |
| SRO7 | 22.63 | <15.00 | S | **7.63** | Lethal(2) giant larvae protein homolog SRO7 OS |
| UBA4 | 22.02 | <15.00 | S | **7.02** | Adenylyltransferase and sulfurtransferase UBA4 OS |
| COA6 | 21.82 | <15.00 | S | **6.82** | Cytochrome c oxidase assembly factor 6 OS |
| SMC1 | 21.60 | <15.00 | S | **6.60** | Structural mainte15ce of chromosomes protein 1 OS |
| CDC8 | 21.59 | <15.00 | S | **6.59** | Thymidylate kinase OS |
| YGR117C | 21.46 | <15.00 | S | **6.46** | Uncharacterized protein YGR117C OS |
| CST6 | 21.30 | <15.00 | S | **6.30** | ATF/CREB activator 2 OS |
| MLF3 | 21.30 | <15.00 | S | **6.30** | Serine-rich protein |
| POL2 | 21.07 | <15.00 | S | **6.07** | DNA polymerase epsilon catalytic subunit A OS |
| SOL4 | 20.79 | <15.00 | S | **5.79** | 6-phosphogluconolactonase 4 OS |
| RCY1 | 20.77 | <15.00 | S | **5.77** | Recyclin-1 OS |
| PSK2 | 20.59 | <15.00 | S | **5.59** | Serine/threonine-protein kinase PSK2 OS |
| HXK1 | 28.56 | 27.20 | 0.000103 | **1.37** | Hexokinase-1 OS |
| YMR196W | 23.99 | 22.71 | 0.005773 | **1.27** | Uncharacterized protein YMR196W OS |
| PGM2 | 27.41 | 26.22 | 0.000040 | **1.18** | Phosphoglucomutase 2 OS |
| NUP192 | 22.88 | 21.88 | 0.041059 | **1.00** | Nucleoporin NUP192 OS |
| KSS1 | <15.00 | 21.34 | S | **-6.34** | Mitogen-activated protein kinase KSS1 OS |
| TCD1 | <15.00 | 21.59 | S | **-6.59** | tRNA threonylcarbamoyladenosine dehydratase 1 OS |
| SGV1 | <15.00 | 21.74 | S | **-6.74** | Serine/threonine-protein kinase BUR1 OS |
| MNN10 | <15.00 | 21.91 | S | **-6.91** | Probable alpha-1,6-mannosyltransferase MNN10 OS |
| FAR8 | <15.00 | 22.24 | S | **-7.24** | Factor arrest protein 8 OS |
