# Supplementary material for: Effects of abolishing Whi2 on the proteome and nitrogen catabolite repression-sensitive protein production
Source: G3 (Bethesda). 2021 Dec 17;12(3):jkab432. doi: 10.1093/g3journal/jkab432 (PMC9210300; doi:10.1093/g3journal/jkab432)
Supplement: jkab432_Supplementary_Table_S17 [file jkab432_supplementary_table_s17.docx]

**Table S-17**

**Proteins whose levels change by an absolute Log_2_ value of equal or greater than 1 when Wild Type (P1) and *whi2*Δ cells (P1-whi2), grown for six Hrs in ME medium, are compared**

| Gene | Log_2_ P1  6 Hr ME | Log_2_ P1-whi2 6 Hr ME | Significance | Log2  P1/P1-whi2 | Function (SGD) |
| --- | --- | --- | --- | --- | --- |
| AFG1 | 23.02 | <15.00 | S | **8.02** | Protein AFG1 OS |
| SOL4 | 22.72 | <15.00 | S | **7.72** | 6-phosphogluconolactonase 4 OS |
| MSC1 | 22.65 | <15.00 | S | **7.65** | Meiotic sister chromatid recombination protein 1 OS |
| GDB1 | 22.49 | <15.00 | S | **7.49** | Glycogen debranching enzyme OS |
| CCP1 | 22.41 | <15.00 | S | **7.41** | Cytochrome c peroxidase, mitochondrial OS |
| YIP4 | 22.39 | <15.00 | S | **7.39** | Interacts with Rab GTPases in late Golgi |
| SSA4 | 22.11 | <15.00 | S | **7.11** | Heat shock protein SSA4 OS |
| PDE1 | 22.08 | <15.00 | S | **7.08** | 3,5-cyclic-nucleotide phosphodiesterase 1 OS |
| SLM1 | 22.02 | <15.00 | S | **7.02** | Phosphatidylinositol 4,5-bisphosphate-binding protein SLM1 OS |
| CBP3 | 21.86 | <15.00 | S | **6.86** | Protein CBP3, mitochondrial OS |
| YAP1 | 21.77 | <15.00 | S | **6.77** | AP-1-like transcription factor YAP1 OS |
| MLF3 | 21.42 | <15.00 | S | **6.42** | Serine-rich protein |
| PSK2 | 20.91 | <15.00 | S | **5.91** | Serine/threonine-protein kinase PSK2 OS |
| TGL4 | 20.29 | <15.00 | S | **5.29** | Lipase 4 OS |
| RNY1 | 20.15 | <150.00 | S | **5.15** | Ribonuclease T2-like OS |
| YGR125W | 19.97 | <15.00 | S | **4.97** | Uncharacterized vacuolar membrane protein YGR125W |
| SAG1 | 24.92 | 20.81 | 8e-05 | **4.10** | Alpha-agglutinin OS |
| GPH1 | 25.94 | 22.73 | 0.0043 | **3.21** | Glycogen phosphorylase OS |
| YGR130C | 22.72 | 19.51 | 0.0015 | **3.21** | Uncharacterized protein YGR130C OS |
| GPM2 | 22.06 | 18.93 | 0.0190 | **3.13** | Phosphoglycerate mutase 2 |
| HXK1 | 28.43 | 26.58 | 6e-06 | **1.86** | Hexokinase-1 OS |
| SSE2 | 24.33 | 22.57 | 0.0126 | **1.76** | Heat shock protein homologue sse2 OS |
| HSP26 | 27.23 | 25.48 | 0.0002 | **1.75** | Heat shock protein 26 OS |
| HSP31 | 22.81 | 21.11 | 0.0158 | **1.70** | Glutathione-independent glyoxalase HSP31 |
| GAD1 | 23.65 | 21.96 | 1e-04 | **1.69** | Glutamate decarboxylase OS |
| YGP1 | 26.89 | 25.38 | 0.0003 | **1.51** | Cell wall-related secretory glycoprotein – induced by nutrient starvation and entry stationary phase |
| YNR034W-A | 23.73 | 22.25 | 2e-05 | **1.48** | Uncharacterized protein YNR034W-A OS |
| GCY1 | 26.45 | 25.03 | 7e-05 | **1.42** | Glycerol 2-dehydrogenase (NADP(+)) OS |
| PGM2 | 27.54 | 26.20 | 0.0001 | **1.36** | Phosphoglucomutase 2 OS |
| UGA2 | 23.32 | 22.04 | 0.0003 | **1.28** | Succinate-semialdehyde dehydrogenase [NADP(+)] OS |
| YPI1 | 23.28 | 22.03 | 0.002 | **1.25** | Type 1 phosphatases regulator YPI1 OS |
| YMR196W | 23.67 | 22.5 | 0.007 | **1.17** | Uncharacterized protein YMR196W OS |
| YML100W | 25.31 | 24.21 | 8e-05 | **1.11** | Trehalose synthase complex regulatory subunit TSL1 OS |
| PET10 | 22.74 | 21.65 | 0.0019 | **1.09** | Perilipin involved in formation and stability of lipid droplets |
| GOR1 | 23.23 | 22.16 | 0.0005 | **1.07** | Glyoxylate reductase 1 OS |
| GSY2 | 25.38 | 24.36 | 0.0004 | **1.02** | Glycogen [starch] synthase isoform 2 OS |
| ENA2 | 21.95 | 22.97 | 0.0021 | **-1.02** | Sodium transport ATPase 2 OS |
| OLE1 | 25.96 | 27.11 | 0.0003 | **-1.16** | Acyl-CoA desaturase 1 OS |
| BUD20 | <15.00 | 20.23 | S | **-5.23** | Bud site selection protein 20 OS |
| UTP30 | <15.00 | 20.32 | S | **-5.32** | Ribosome biogenesis protein UTP30 OS |
| YCR102C | <15.00 | 21.12 | S | **-6.12** | Uncharacterized protein YLR460C OS |
| ELP6 | <15.00 | 21.43 | S | **-6.43** | Elongator complex protein 6 OS |
| NST1 | <15.00 | 21.70 | S | **-6.70** | Stress response protein NST1 OS |
| FLC2 | <15.00 | 21.76 | S | **-6.76** | Flavin carrier protein 2 OS |
| IRC22 | <15.00 | 22.24 | S | **-7.24** | Increased recombination centers protein 22 OS |
| ECM15 | <15.00 | 22.47 | S | **-7.47** | UPF0045 protein ECM15 OS |
| BUD32 | <15.00 | 22.48 | S | **-7.48** | EKC/KEOPS complex subunit BUD32 OS |
